# Dysregulated lipid metabolism in a retinal pigment epithelial cell model and serum of patients with age-related macular degeneration

**DOI:** 10.1186/s12915-025-02198-8

**Published:** 2025-04-12

**Authors:** Ana Álvarez-Barrios, Lydia Álvarez, Pilar Sáenz de Santa María, Montserrat García, Jorge R. Álvarez-Buylla, Rosario Pereiro, Héctor González-Iglesias

**Affiliations:** 1 Fundación de Investigación Oftalmológica, Oviedo, Spain; 2 Department of Physical and Analytical Chemistry, University of Oviedo, Oviedo, Spain; 3 Instituto Oftalmológico Fernández-Vega, Oviedo, Spain; 4 Instituto de Productos Lácteos de Asturias, Consejo Superior de Investigaciones Científicas (IPLA-CSIC), Oviedo, Spain

**Keywords:** Multiomics, Ageing, Age-related macular degeneration, Lipid metabolism, Retinal pigment epithelium, Serum

## Abstract

**Background:**

Age-related macular degeneration (AMD) is a leading cause of blindness, characterized by retinal pigment epithelium (RPE) dysfunction, extracellular deposit formation, and disrupted lipid metabolism. Understanding the molecular changes underlying AMD is essential for identifying diagnostic markers and therapeutic targets.

**Results:**

This multiomic study employed a primary RPE culture model to investigate age-related changes associated with AMD. Over 25 weeks, RPE cells exhibited phenotypic deterioration, including depigmentation, cell shape deformation, and barrier integrity loss, accompanied by extracellular deposit formation. Transcriptomic analysis revealed dysregulation of genes involved in lipid metabolism, including pathways for cholesterol transport, glycerophospholipids, and ceramide biosynthesis. Metabolomic profiling further identified significant changes in glycerophospholipid and sphingolipid metabolism, highlighting a decline in phospholipid species and ceramide accumulation. Serum analysis of AMD patients revealed altered levels of 18 lipids identified in RPE cultures. Four lipids showed significant differences compared to controls: GlcCer(d16:1/18:0) (1.23-fold increase, adj. *p* value < 0.001), PE(19:1(9Z)/22:2(13Z,16Z)) (0.34-fold decrease, adj. *p* value < 0.001), PE(15:0/20:3(5Z,8Z,11Z)) (0.66-fold decrease, adj. *p* value < 0.05), and PC(22:2(13Z,16Z)/13:0) (0.71-fold decrease, adj. *p* value < 0.05). These findings underscore the systemic nature of lipid dysregulation in AMD and the translational relevance of the RPE model.

**Conclusions:**

This study highlights the significant role of lipid metabolism dysregulation in AMD pathogenesis. The consistent lipidomic alterations observed in RPE cultures and AMD patient serum reinforce their potential as biomarkers for disease progression and therapeutic targets. These findings provide a robust framework for understanding AMD-associated lipid metabolism changes and their systemic impact.

**Supplementary Information:**

The online version contains supplementary material available at 10.1186/s12915-025-02198-8.

## Background

Age-related macular degeneration (AMD) is a leading cause of blindness, affecting over 200 million people worldwide [1]. Its progression is associated with significant changes in the retinal pigment epithelium (RPE), which plays an important role in vision by supporting the function of the highly active photoreceptor cells of the retina. The RPE facilitates the recycling of the photoreceptor pigments and damaged cellular material, while regulating the transport of nutrients and waste products between the photoreceptors and the blood vessels within the choroid [2]. As the visual system ages, natural deterioration leads to functional alterations, including impaired retinal cell activity. These age-related changes in eye function significantly increase the risk of developing vision-related disorders. Early signs of AMD include the abnormal accumulation of drusen, extracellular deposits rich in proteins, lipids, and minerals, in the space between the macular RPE and the Bruch’s membrane. These deposits disrupt nutrient and oxygen exchange, leading to RPE dysfunction and photoreceptor degeneration [3].

Despite extensive research, the transition from healthy ageing to AMD remains poorly understood, largely due to the complex interplay of genetic, metabolic, and environmental factors. Traditional studies often focus on understanding the molecular processes that could potentially play a part on AMD pathogenesis, such as oxidative stress and mitochondrial deterioration [4], lysosomal dysfunction and altered proteostasis [5], abnormal autophagy [6], cellular senescence [7], inflammation [8], or lipid dyshomeostasis [9–11], limiting our knowledge about the multifactorial nature of AMD. Fingerprinting the molecular processes of progressive RPE deterioration could help identify the risk factors contributing to the transition from non-pathological ageing, which may involve mild visual decline, to early AMD, which is characterized by specific pathological changes, including sub-RPE deposit formation, drusen accumulation, and disruptions in lipid metabolism.

Recent advances in omics technologies, including transcriptomics and metabolomics, provide powerful tools to comprehensively analyze the molecular changes underlying AMD pathogenesis. These multiomic approaches allow the integration of large datasets to identify novel biomarkers and therapeutic targets [12, 13]. In vitro models of RPE cells, which can mimic the formation of extracellular deposits and RPE atrophy, represent an essential first step in identifying and understanding the molecular processes and potential biomarkers involved in early AMD pathogenesis, without influences of external variables [14–16]. Despite their potential, studies applying multiomics to RPE models of AMD remain scarce. This study addresses this gap by utilizing a long-term primary RPE culture model to investigate temporal changes in gene expression and metabolism. Our hypothesis is that prolonged cultivation of RPE cells, i.e., 6 months, induces age-related molecular changes, recapitulating early AMD pathology, particularly dysregulation in lipid metabolism. By integrating transcriptomic and metabolomic approaches with targeted protein analysis, we aim to identify key pathways contributing to RPE dysfunction. Furthermore, we investigate the relevance of these findings by observing systemic changes in AMD patients, with a focus on identifying altered serum lipids as potential biomarkers.

## Results

### Modification of RPE characteristics

#### Pigmentation and morphology

**Fig. 1:**
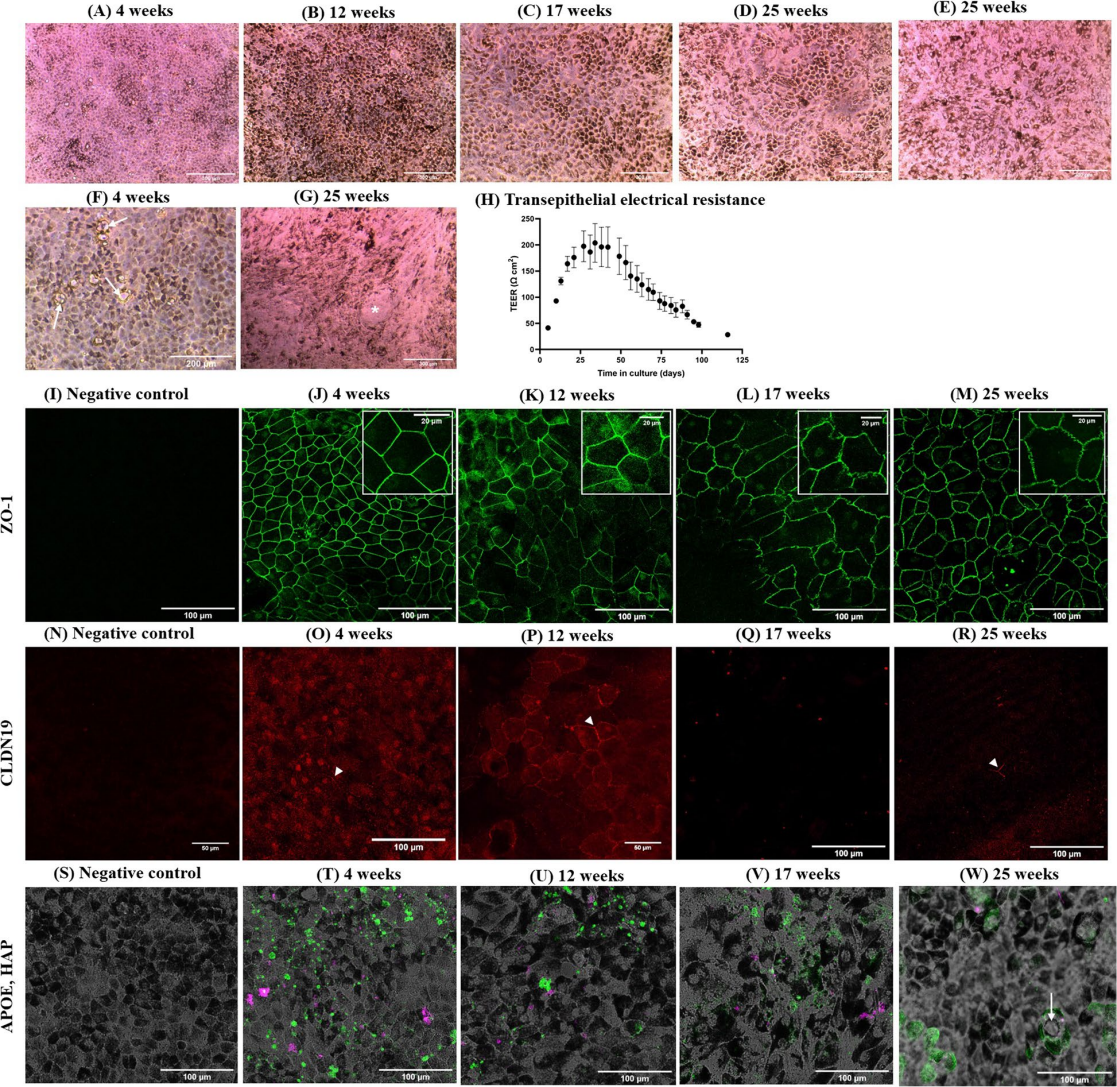
**A**–**G** Optical microscopy images showing the pigmentation and morphology of cells in culture at 4 (**A**, **F**), 12 (**B**), 17 (**C**), and 25 weeks (**D**–**E**). Intra-well heterogeneity was high after longer times in culture, appearing differences in cell phenotype across the well surface, like typical RPE (**D**), rounded shapes (**E**), or fibroblast-like cells (**G**). Potential extracellular deposits in cell cultures of 4 weeks are shown as arrows (**F**), while asterisk (*) points to a gap in the cell monolayer in **G**. **H** Evolution of transepithelial electrical resistance (TEER) with time (*n* = 9). Data points represent mean value of TEER expressed in Ω cm^2^ and error bars, 95% CI. **I**–**M** Immunolocalization of ZO-1 in cultures of 4, 12, 17, and 25 weeks. A zoomed image of ZO1 signal in RPE cell membranes shows the progression from clean to frizzled over time. **N**–**R** Immunolocalization of CLDN19 in cultures of 4, 12, 17, and 25 weeks. Arrowheads in CLDN19 panel point to signals on the cell membrane. **S**–**W** Localization of extracellular deposits formed by ApoE (green) and HAP (magenta) in the cell cultures over time through immunocytochemistry, where arrow points to a potential deposit between RPE cells

Cells in culture acquired typical RPE characteristics, such as a cobblestone shape and pigmentation granules over time. These features were noticeable at 4 weeks (Fig. 1A) and became more pronounced at 12 weeks (Fig. 1B). However, these characteristics began to be lost after 17 weeks of culture in several areas of the insert surface, with a decrease in pigmentation and an increase in cells with rounded shapes, rather than polygonal ones (Fig. 1C). These changes were more pronounced at 25 weeks, when depigmented, rounded cells (Fig. 1E) and fibroblast-like cells (Fig. 1G) were frequently observed, alongside still healthy-looking RPE cells (Fig. 1D) and gaps in the epithelium (Fig. 1G). Phenotypic variability was very high at late time points, with significant differences between cells grown at the edges and those at the center of the wells.

#### Barrier function

The integrity of the cell monolayer was evaluated by measuring the transepithelial electrical resistance (TEER) of the cultures throughout the experiment. Mean TEER values are shown in Fig. 1H. TEER progressively increased from 41 ± 1.75 (95% CI) Ω cm^2^ at the beginning of the culture (5 days) to a maximum value of 217 ± 46.35 (95% CI) Ω cm^2^ after 34 days. From days 34 to 53, TEER remained constant, but then began to decrease steadily, reaching values lower than the initial measurements at 116 days (28 ± 4.50 (95% CI) Ω cm^2^). Strong cell–cell junctions are essential for epithelial cells to form a robust cellular barrier. To evaluate the cohesion of the epithelium and the polarization of cells over time, two proteins that form tight junctions—zonula occludens-1 (ZO-1) and claudin 19 (CLDN19)—were studied in the RPE cell cultures. As shown in Fig. 1I–M, ZO-1 is present in the RPE cultures at all time points, but with morphological differences. At 4 and 12 weeks in culture, ZO-1 forms a uniform band across the membrane of cells. However, with prolonged culture time (17 and especially 25 weeks), the ZO-1 signal becomes more disorganized and frizzled. In the case of CLDN19 (Fig. 1N–R), the protein is most easily detected at 12 weeks in culture, where it appears in the membrane of most RPE cells, but less detectable at earlier or later time points.

#### Extracellular deposition of lipid-transporting apolipoprotein E and hydroxyapatite

Formation of drusen-like deposits occurred in the cellular model of RPE, which was visible through optical microscopy (Fig. 1F). Additionally, fluorescent antibodies and probes targeting two compounds commonly found in drusen, apolipoprotein E (APOE) and hydroxyapatite (HAP), were used to further assess the deposits (Fig. 1S–W). APOE and HAP were detected in the extracellular space of cell cultures at all time points, often in close proximity and occasionally co-localizing.

### Changes on gene expression by transcriptomics

#### Regression and pathway enrichment analysis to identify genes associated with time in culture

**Fig. 2:**
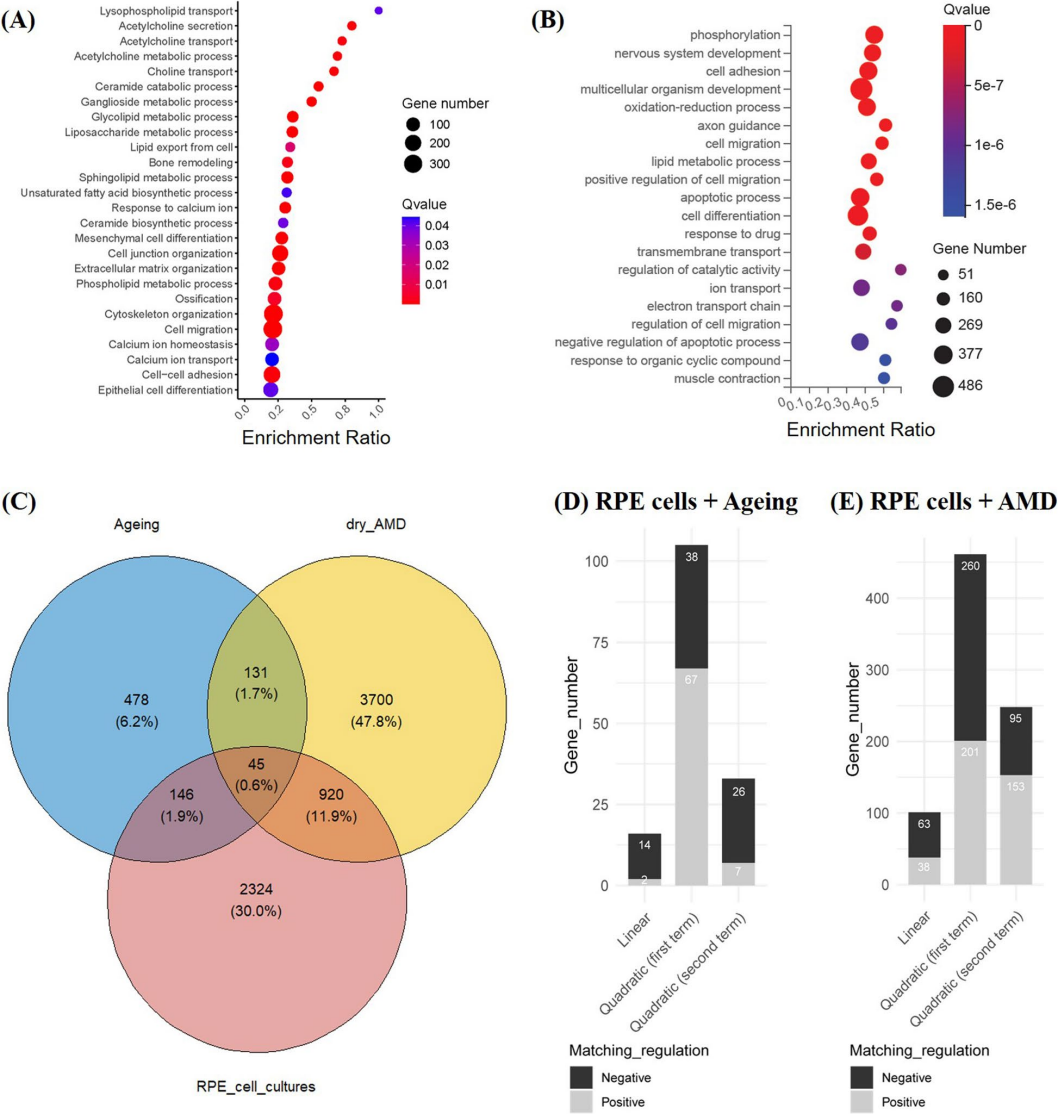
Bubble charts of enriched biological processes in RPE cell cultures and comparison with in vivo obtained sample data. **A** Enriched biological processes identified from genes significantly associated with time in culture. Each process is represented by a bubble on the Y-axis. Bubble size reflects the number of SCGs annotated to the corresponding GO term, while color indicates the significance of enrichment (*q* value). The X-axis shows the enrichment ratio (number of SCGs annotated to the GO term divided by the total number of genes annotated to that GO term). **B** Top 20 most significantly enriched biological processes raised by DEGs between 25 (*n* = 2) and 4 weeks (*n* = 3) in culture. **C** Venn diagram showing overlap of time-associated genes in RPE cell cultures with genes from ageing dataset (Ref. GSE159435) and dry AMD dataset (Ref. 10.5281/zenodo.7020215). **D** Genes exhibiting similar expression patterns over time when comparing RPE cell cultures and donor age (“Ageing” dataset). **E** Genes showing similar expression trends over time in RPE cell cultures and AMD diagnosed patients (“AMD” dataset). Where applicable, the analyses included data from three biological replicates of cell cultures at 4, 12, and 17 weeks, along with two additional replicates at 25 weeks. Additionally, the dataset comprised 13 replicates of RPE samples from the “AMD” dataset and 85 replicates of macular RPE/choroid from “Ageing” dataset

Implementing regression models to transcriptomic data using R (v4.4.2) allowed the identification of 3435 significantly correlated genes (SCGs) with time in culture. Linear regression model fitting identified 884 genes exhibiting linear expression patterns over time, while 2551 genes displayed a bell-shaped temporal pattern, fitting a quadratic regression model. The analysis of biological processes based on SCGs identified a total of 1000 significantly altered Gene Ontology (GO) terms. Noteworthy clusters of GO terms related to lipid metabolism, epithelial-to-mesenchymal transition (EMT), and calcium deposition were identified (Additional file 1: Table S1). Figure 2A provides representative biological processes from these categories. Within lipid metabolism, GO terms such as sphingolipid metabolic process (GO:0006665), glycolipid metabolic process (GO:0006664), and ceramide biosynthesis and catabolism (GO:0046513, GO:0046514) were significantly altered, indicating age-associated disruptions in lipid homeostasis. Other affected lipid-related processes included acetylcholine and lysophospholipid transport, highlighting changes in lipid trafficking mechanisms. Biological processes related to EMT, such as cytoskeleton organization (GO:0007010), cell migration (GO:0016477), mesenchymal differentiation (GO:0048762), and extracellular matrix remodeling (GO:0030198), suggest that these processes were progressively dysregulated during the culture period. Furthermore, alterations in calcium-related biological processes, including ossification (GO:0001503), calcium ion homeostasis (GO:0055074), and calcium ion transport (GO:0006816), reflected significant changes in calcium regulation over time.

Individual comparisons of gene expression between 4 and 25 weeks in culture by DESeq2 method and Dr. Tom platform revealed differentially expressed genes (DEGs) that included those that did not fit a linear nor quadratic regression model with time. Pathway enrichment analysis conducted with DEGs further elucidated temporal changes in gene expression between 4 and 25 weeks of culture (Fig. 2B; Additional file 1: Fig. S1 provides additional comparisons). Molecular processes such as phosphorylation (GO:0016310) and oxidation–reduction (GO:0055114) were significantly enriched after 25 weeks in culture, together with regulatory processes related to catalytic activity (GO:0050790), apoptosis (GO:0043066), gene expression (GO:0010628), and post-translational protein modification (GO:0043687). Enrichment of processes like response to ischemia (GO:0002931) and cellular response to hypoxia (GO:0071456) suggests oxidative stress, while enrichment of transmembrane transport (GO:0055085), ion transport (GO:0006811), electron transport chain (GO:0022900), protein transport (GO:0015031), and ion transmembrane transport (GO:0034220) processes highlights the alteration of molecule transport across cell membranes, especially of ions. GO terms linked to lipids, calcium deposition, and EMT were also enriched at 25 weeks when compared to 4 weeks, matching the results derived from SCGs.

#### *Comparison of *in vitro* data with published datasets of real samples*

SCGs identified through either linear or quadratic regression models were compared with expression datasets from real samples to evaluate the similarity between time-associated transcriptomic changes in the RPE cell culture model and those occurring during ageing or AMD pathogenesis in vivo. Two published datasets were used for comparison: the “Ageing” dataset, containing expression data from 13 RPE samples isolated from donors aged 31 to 93 years [17], and the “AMD” dataset, containing expression data from macular RPE/choroid samples derived from 36 healthy controls and 35 dry AMD patients [18]. In the “Ageing” dataset, the authors identified 709 genes that associated with age through a linear regression model, and we extended this by including 91 genes with significant quadratic regression fits, accounting for sequencing batch and sex as covariates, resulting in 800 SCGs associated with ageing. In the “AMD” dataset, 4796 DEGs were identified between dry AMD patients and healthy controls, with data encompassing both RPE and choroid tissue.

A Venn diagram (Fig. 2C) was used to identify overlapping genes between the RPE cell culture model, the “Ageing” dataset, and the “AMD” dataset. The overlap analysis revealed 965 genes shared between the RPE cell cultures and the “AMD” dataset, of which 810 exhibited similar expression trends (Fig. 2E), while 191 genes overlapped with the “Ageing” dataset, with 154 showing similar trends (Fig. 2D). Enrichment analysis of the 810 shared genes between the RPE cultures and the “AMD” dataset revealed significant alterations in processes such as calcium deposition, lipid metabolism, and protein homeostasis (data not shown).

#### Differentiation state of RPE cells over a 6-month follow-up

The expression of genes associated with RPE identity was studied through regression analysis and differential expression comparisons between 4, 12, 17, and 25 weeks in culture to evaluate the differentiation state of cells. Genes specific to the visual cycle, including bestrophin 1 (*BEST1*), retinol dehydrogenase 5 (*RDH5*), lecithin retinol acetyltransferase (*LRAT*), RPE-retinal G-coupled receptor (*RGR*), retinal pigment epithelium-specific 65 kDa protein (*RPE65*), and retinaldehyde binding protein (*RLBP1*), exhibited increased expression until 17 weeks, followed by a pronounced decline at 25 weeks. Similarly, genes involved in melanin biosynthesis, such as tyrosinase (*TYR*), dopachrome tautomerase (*DCT*), tyrosinase-related protein 1 (*TYRP1*), and melanocyte inducing transcription factor (*MITF*), reached peak expression at 4 weeks, maintained relatively high levels through 17 weeks, and then experienced a sharp decrease by 25 weeks (Additional file 1: Fig. S2).

#### Transcriptomic alterations with implications in RPE ageing, lipid metabolism dysregulation, and AMD pathogenesis

**Fig. 3:**
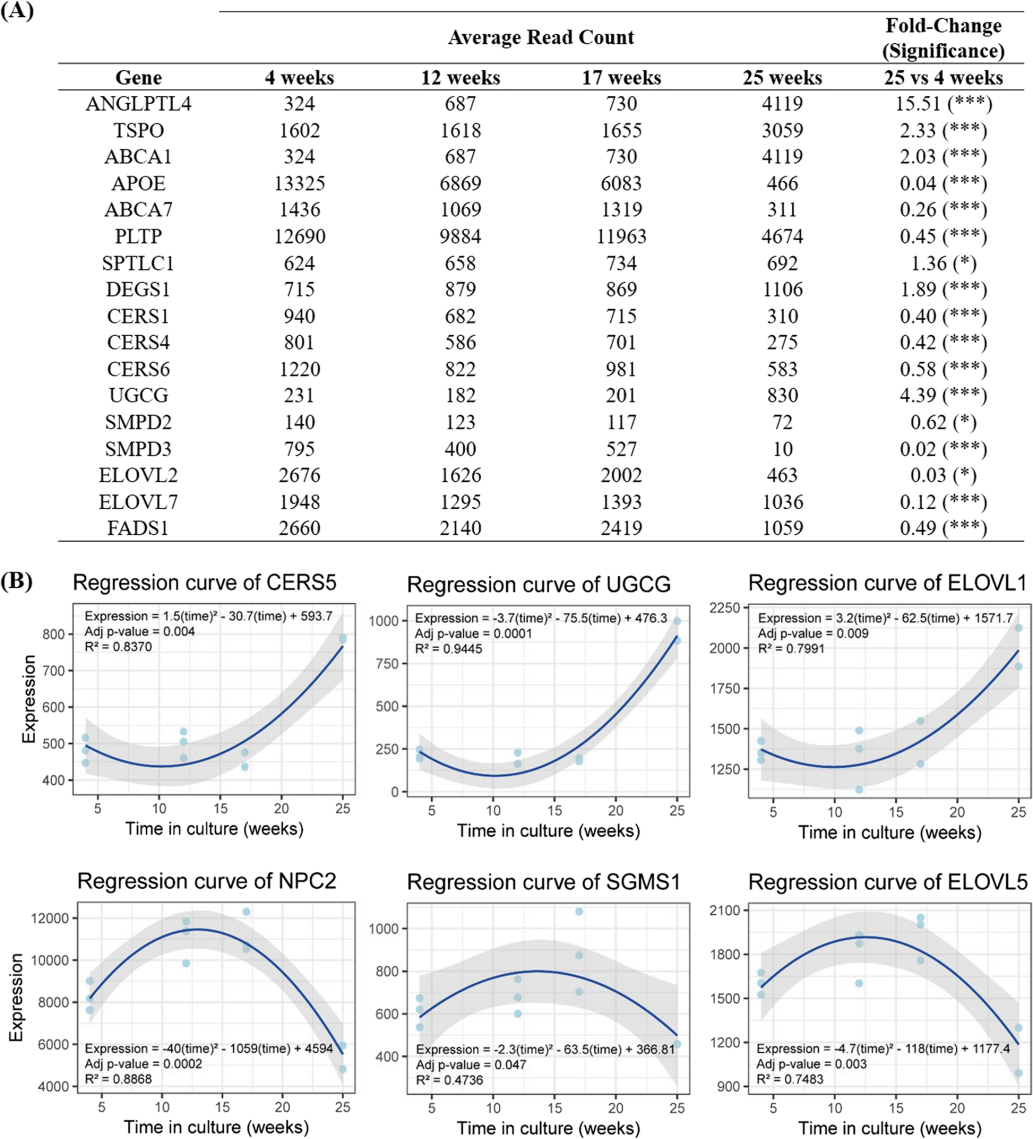
Temporal changes of genes associated with lipid metabolism. **A** DEGs identified between 4 (*n* = 3) and 25 (*n* = 2) time points (see Additional file 3 for individual data values). Fold-changes and statistical significance were obtained by DESeq2 method. ns: *q* value > 0.05; *: *q* value < 0.05; **: *q* value < 0.01; ***: *q* value < 0.001. **B** SCGs identified through linear and quadratic regression models. Analyses included data from 3 biological replicates of the cell cultures of 4, 12, and 17 weeks and 2 replicates of 25 weeks (see Additional file 3 for individual data values). Equation of the regression curve is shown together with the associated coefficient of determination (*R*^2^) and *p* value adjusted by Benjamini–Hochberg method

Figure 3 shows temporal expression changes of genes associated with lipid metabolism processes implicated in AMD pathogenesis identified as SCGs or DEGs between 4 and 25 weeks in culture. Significant dysregulation of lipid metabolism was observed with the age of our RPE cultures. After 6 months in culture, changes of genes involved in cholesterol metabolism included upregulation of angiopoietin-like 4 (*ANGPTL4*), translocator protein (*TSPO*), and ATP binding cassette subfamily A member 1 (*ABCA1*) and downregulation of *APOE*, ATP binding cassette subfamily A member 7 (*ABCA7*), and phospholipid transfer protein (*PLTP*). Niemann-Pick disease intracellular cholesterol transporter 2 (*NPC2*) was also downregulated by the 25th week in culture in comparison to its initial expression levels but was substantially upregulated at 12 and 17 weeks in culture, describing a concave curve with time. Dysregulated genes involved in sphingolipid metabolism included serine palmitoyltransferase long chain base subunit 1 (*SPTLC1*), delta 4-desaturase sphingolipid 1 (*DEGS1*), and ceramide synthase 5 (*CERS5*), with increased expression, and ceramide synthases 1 (*CERS1*), 4 (*CERS4*), and 6 (*CERS6*) whose expression decreased. Additionally, gene expression of sphingomyelin synthase 1 (*SGMS1*), involved in sphingomyelin (SM) synthesis from ceramides, followed a bell-shape trend with time by being slightly upregulated at the mid-point of the cultivation period and downregulated at the end of the 25 weeks in culture. Expression of UDP-glucose ceramide glucosyltransferase (*UGCG*), involved in glucosylceramide synthesis, one of the two types of hexosylceramides, was upregulated, while genes encoding sphingomyelinases 2 (*SMPD2*) and 3 (*SMPD3*) that synthesize ceramide by hydrolysis of SM were downregulated. In relation with fatty acid metabolism, temporal expression of the gene family of fatty acid elongases (*ELOVL*) showed downregulation of three out of the seven *ELOVL* (*ELOVL 2*, *5*, and *7*) genes and upregulation of *ELOVL1* after the 6 months in culture. *ELOVL5* was transiently upregulated between 12 and 17 weeks in culture, whereas *ELOVL1* was transiently downregulated at 12 weeks. Moreover, expression of fatty acid desaturase 1 (FADS1), involved in polyunsaturated fatty acid biosynthesis, was downregulated at 25 weeks.

### Untargeted metabolomic analysis

**Fig. 4:**
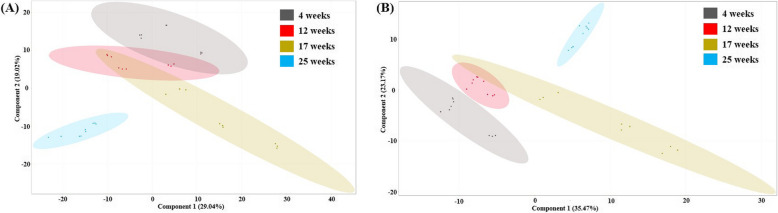
Score scatter plot of the PCA model for RPE cells cultured during 4 (gray, *n* = 3 biological replicates, each measured 3 times), 12 (red, *n* = 3 biological replicates, each measured 3 times), 17 (beige, *n* = 3 biological replicates, each measured 3 times), and 25 (blue, *n* = 3 biological replicates, each measured 3 times) weeks after LC–MS analysis in positive (**A**) and negative (**B**) ionization modes (see Additional file 4 for individual data values). X-axis and Y-axis reveal the scores of the first and the second component, respectively. The principal components 1, 2, and 3 (PC1, PC2, and PC3) could explain 57% of total variance. Points of different colors represent run samples from each experimental group (3 analytical replicates and 3 biological replicates) and ellipses, the 95% confidence region

Cellular samples from 4, 12, 17, and 25 weeks were analyzed by liquid chromatography-mass spectrometry (LC–MS) in both positive and negative ionization modes to maximize metabolome coverage, resulting in a total of 634 and 252 distinct molecular features, respectively. Unsupervised principal component analysis (PCA) was performed using Mass Profiler Professional (MPP) software to assess the similarity between time points, distinguishing the four experimental groups corresponding to 4, 12, 17, and 25-week-old RPE cells (Fig. 4). Principal components 1 and 2 (PC1 and PC2) explained 48% of the variance in the positive ionization mode and 58% in the negative ionization mode. A mean comparison test using Kruskal–Wallis method revealed 498 significantly different metabolites among time points (adjusted *p* value < 0.05) in positive ionization mode and 222 in negative ionization mode. To refine the results, an additional filter was applied to select metabolites with a fold-change > 2, leading to 283 and 100 features in the positive and negative ionization modes, respectively (Additional file 1: Fig. S3 shows the heatmap of hierarchical clustering analysis of differential metabolites). These compounds were tentatively identified using ID browser, resulting in 185 distinct molecules in the positive mode and 68 in the negative mode. Normalized, averaged data for each of the tentatively identified compounds are provided in Additional file 2: Tables S2 and S3. Compound identities were further confirmed through LC–MS/MS analysis.

**Fig. 5:**
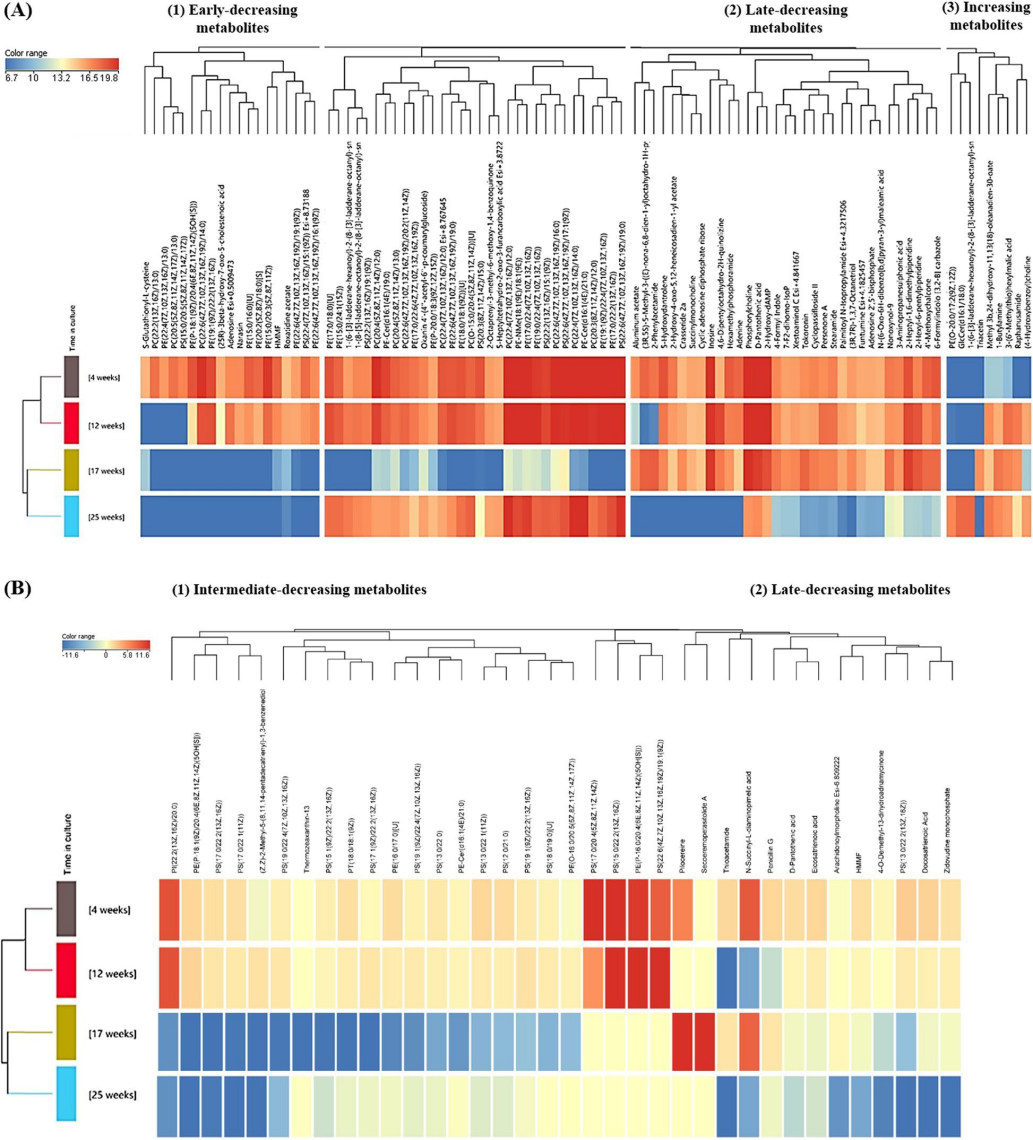
Heatmap of hierarchical clustering analysis of differential metabolites for 25 weeks (*n* = 3 biological replicates, each measured 3 times) old RPE cells versus 4 (*n* = 3 biological replicates, each measured 3 times), 12 (*n* = 3 biological replicates, each measured 3 times), and 17 (*n* = 3 biological replicates, each measured 3 times) weeks old cells, **A** in positive ion mode and **B** in negative ion mode (see Additional file 4 for individual data values). Significantly different compounds were found through Kruskal–Wallis test with Benjamini–Hochberg correction and only the ones with adjusted *p* value < 0.001 and fold-change > 16 were plotted. Data was averaged among replicates. Relevant clusters of metabolites are highlighted

The data were subjected to stricter statistical and biological significance thresholds (adjusted *p* value < 0.001 and fold-change > 16) for hierarchical clustering analysis based on Euclidean distance calculation for both metabolites and experimental groups (Fig. 5). The sample groups clustered into two main pairs, corresponding to the earlier time points (4 and 12 weeks) and the later time points (17 and 25 weeks). Inspection of metabolites in positive ion mode (Fig. 5A) revealed three clusters of compounds with distinct time-associated patterns relevant to the study: (1) metabolites decreasing early (from 12 weeks onwards), including S-glutathionyl-L-cysteine, adenosine, and several phospholipids of the family of the phosphatidylethanolamines, phosphatidylcholines, and phosphatidylserines; (2) metabolites decreasing later in time (at 25 weeks), including 2-phenylacetamide, phosphorylcholine, adenine, 2-hydroxy-dAMP, and palmitoyl N-isopropylamide; and (3) metabolites increasing with time, including PE(O-20:0/17:2(9Z, 12Z)), GlcCer(d16:1/18:0), and triacetin. In negative ion mode (Fig. 5B), two main types of metabolites were distinguished: (1) metabolites decreasing at intermediate time (i.e., 17 weeks) and (2) metabolites decreasing later in time (i.e., 25 weeks), including phosphatidylethanolamines and phosphatidylserines.

### Integrative analysis of transcriptomic and metabolomic changes over time

**Fig. 6:**
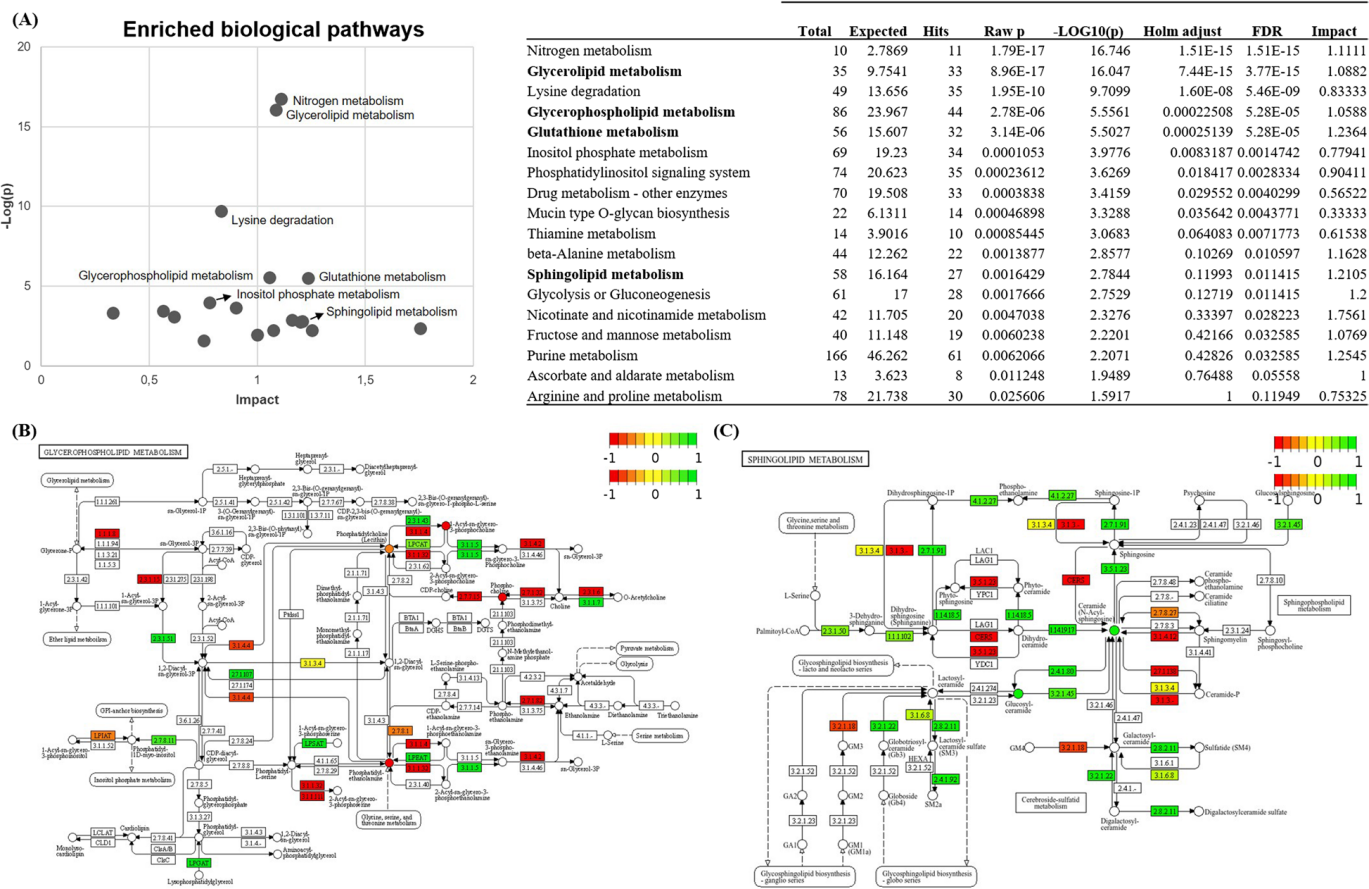
**A** Joint pathway analysis combining data from transcripts (*n* = 3) and metabolites (*n* = 3 biological replicates, each measured 3 times) showing statistically significant differences between 25 and 4 weeks in culture (see Additional files 3 and 4 for individual data values). Integration of transcriptomic and metabolomic data using Metaboanalyst (metaboanalyst.ca) and KEGG pathway database. Significance of pathway enrichment was calculated through query combination integration method and impact was estimated through degree centrality topology measurement. Pathways of high statistical significance (Holm adjusted *p* value < 0.01) are highlighted. **B** Glycerophospholipid (KEGG id: hsa00564) and **C** sphingolipid (KEGG id: hsa00600) metabolic pathway changes in RPE cells at the end (25 weeks) and beginning of the experiment (4 weeks in culture) using Pathview (pathview.uncc.edu). Edges show interactions between significant metabolites (adjusted *p* value < 0.05; circles) and DEGs (*q* value < 0.05; squares) involved in the metabolism of glycerophospholipids and sphingolipids detected in the cell samples. Red and green color of nodes indicate down or upregulation, respectively, in the RPE cells of 25 weeks when compared with cells of 4 weeks. Undetected genes and metabolites on the cell samples appear in white

A joint pathway analysis was conducted by integrating transcriptomic and metabolomic data using Metaboanalyst (metaboanalyst.ca) and KEGG pathway database. Figure 6A highlights the statistically significant enriched pathways in RPE cells in culture when comparing 25 and 4-week time points. This analysis was performed using the query combination integration method, with pathway impact estimated through degree centrality topology measurement. The most categorical pathways identified include nitrogen metabolism, glycerolipid metabolism, lysine degradation, glycerophospholipid metabolism, and glutathione metabolism (Holm adjusted *p* value < 0.01). Additional joint pathway analysis and graphical representation were conducted using Pathview (pathview.uncc.edu) and the KEGG pathway database. Figure 6B and C illustrate changes in the glycerophospholipid and sphingolipid metabolic pathways, respectively, in RPE cells at 25 weeks compared to 4 weeks. Transcripts are represented as squares, while metabolites, as circles in the pathway diagrams.

### Relative quantification of lipids in AMD patients and control subjects

The study included a total of 40 participants (20 dry AMD patients and 20 controls), with no significant differences in sex, age, or comorbidities, including dyslipidemia, between groups (Additional file 2: Table S4). A targeted method was developed and optimized for the relative quantitative analysis of selected molecular compounds that exhibited significant temporal changes in cultured RPE cells. These compounds were analyzed in the serum of AMD patients and control subjects using LC–MS. Eighteen of the 23 molecules identified as significantly different in RPE cells over time were successfully quantified in serum, the majority of which were lipids. Internal deuterated lipid standards were used to ensure accurate normalization and quantification of lipid species. Normalized lipid levels for both control and AMD groups are provided in Additional file 2: Table S5.

**Fig. 7:**
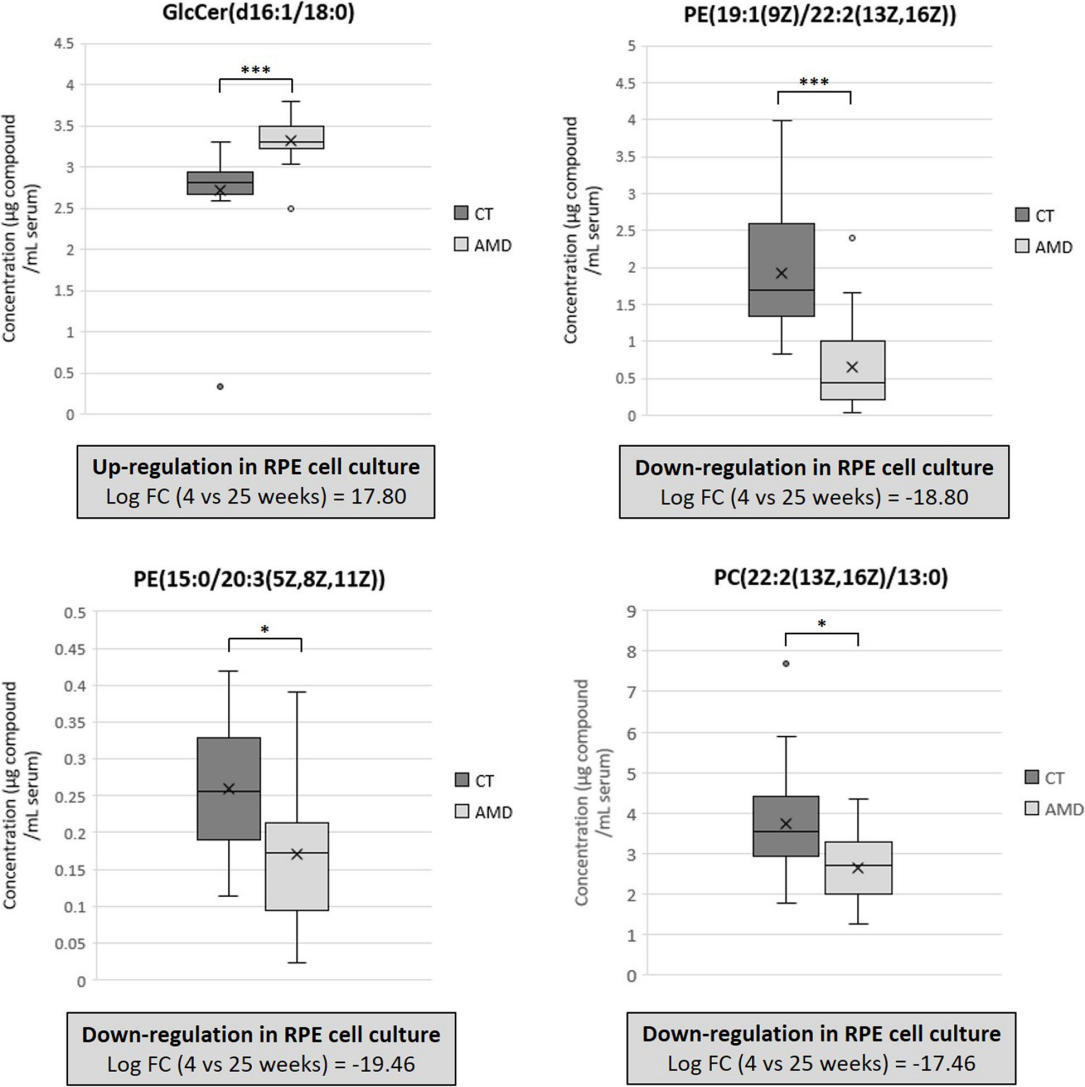
Box plots of lipids showing statistical significance (adjusted *p* value < 0.05) between control (*n* = 20) subjects and AMD (*n* = 20) patients. Statistical significance was calculated through Mann–Whitney [GlcCer(d16:1/18:0), PE(19:1(9Z)/22:2(13Z,16)), and PC(22:2(13Z,16Z)/13:0)] or unpaired *t*-test [PE(15:0/20:3/5Z,8Z,11Z)] with Benjamini–Hochberg multiple testing correction and is shown in the graphs as asterisks (*: adjusted *p* value < 0.05; **: adjusted *p* value < 0.01; ***: adjusted *p* value < 0.001). Regulation of the same metabolites in the RPE cell cultures is shown as the comparison between 4 (*n* = 3 biological replicates, each measured 3 times) and 25 weeks (*n* = 3 biological replicates, each measured 3 times) in culture below each graph (see Additional file 4 for individual data values)

Figure 7 presents box plots of the lipids exhibiting statistically significant differences between control subjects and AMD patients (adjusted *p* value < 0.05, Benjamini–Hochberg correction for multiple comparisons). The Mann–Whitney test was used for statistical analysis of GlcCer(d16:1/18:0), PE(19:1(9Z)/22:2(13Z,16Z)), and PC(22:2(13Z,16Z)/13:0), while the unpaired *t*-test was applied to PE(15:0/20:3(5Z,8Z,11Z)). The regulation of these metabolites in RPE cell cultures over time is also shown below each respective graph, comparing data from 4 and 25 weeks in culture. Notably, the regulatory changes observed in these metabolites were consistent between the serum of AMD patients and RPE cells after 25 weeks in culture, underscoring the potential translational relevance of these findings.

## Discussion

Ageing is the major risk factor for AMD, leading to progressive changes in RPE cells such as alterations of melanin granules, accumulation of lipofuscin, lipid metabolism dysfunction, atrophy of cytoplasmic projections, and the formation of extracellular deposits. However, the molecular mechanisms involved in the transition from healthy ageing to AMD remain under active research. This study employed a multiomic approach to investigate long-term primary cultures of human RPE cells, which produce abnormal extracellular deposits due to the physical barrier created by the culture system, impeding the regular movement of secreted material, as a model of in vitro drusen-in-a-dish system. There is significant interest in developing reliable cell and tissue culture models for AMD research due to their potential as high-throughput, cost-effective, and manipulable systems. These models are especially valuable for studying the early stages of drusen formation and screen potential therapeutic compounds. Primary human fetal RPE cells, in particular, are ideal for examining AMD-associated genetic and physiological changes, as well as identifying candidate biomarkers [19–23]. Sub-RPE deposits formed by primary RPE monolayers in vitro closely resemble human AMD drusen in composition, forming spontaneously and consistently within weeks, much faster than the decades required for drusen formation in vivo. In this study, we examined the transcriptomic and metabolomic alterations in RPE cells over time, mimicking the rise of AMD risk factors associated with ageing. Furthermore, we extended our findings to a systemic level by evaluating a set of compounds identified in the RPE AMD model in the serum of AMD patients and control subjects.

### RPE cells acquire a deteriorated phenotype after the fourth month in culture

After 1 month in culture, primary fetal RPE cells developed phenotypic characteristics typical of mature RPE, such as hexagonal shape and dark pigmentation. Pigment density continued to increase over time until the 12th week in culture. However, after 17 weeks, pigmentation progressively decreased in a heterogeneous manner across the insert surface. Greater depigmentation was observed at the interface between the center and borders of the well (Fig. 1A–G), suggesting a decrease in melanosomes over time [24]. RPE pigmentation is critically linked to retinal development and provides protective benefits against AMD. The incidence of AMD varies significantly across populations, with individuals of European descent affected 8.1 times more frequently than other groups [25]. In our study, the loss of pigmentation was accompanied with cell shape deformation, with a mix of hexagonal, rounded, and elongated cells observed in cultures at 25 weeks. Generally, central cells maintained characteristic RPE features longer than peripheral cells, though also showed signs of degeneration by the end of the study.

The integrity of the cell barrier followed a pattern similar to that of cell morphology and pigmentation. TEER, which measures the resistance of the cell monolayer to ionic currents, was estimated to be approximately 206 ± 151 Ω cm^2^ for the fetal RPE and 79 ± 48 Ω cm^2^ for the adult RPE in vivo, noting that TEER values can vary significantly depending on the donor and culture conditions [26]. The TEER values observed for RPE cells in vitro at 4 and 12 weeks of culture were comparable to those of fetal and adult RPE in vivo, respectively (Fig. 1H). This was accompanied by the formation of tight junctions, with ZO-1 protein at 4 weeks (Fig. 1J) and CLDN19 after 12 weeks in culture (Fig. 1P), indicating strong cohesion of the cellular monolayer. However, the high integrity observed at 4 and 12 weeks in culture declined at later time points. By 17 weeks, TEER levels became negligible, and by 25 weeks, CLDN19 signal was absent, while ZO-1 exhibited a disrupted, frizzled distribution on the cell membranes (Fig. 1R), signaling a loss of barrier function.

From 4 weeks in culture onwards, RPE cells displayed the ability to produce extracellular deposits containing APOE and HAP (Fig. 1S–W), an early sign of cellular dysfunction and strongly associated with early AMD. Optical microscopy revealed distinct bubble-like structures, with abundant dot-like extracellular signals of APOE and HAP throughout the cultivation time. Spontaneous regression of drusen has been reported as part of its natural progression in the retina [27], often associated with late-stage AMD [28].

### Time-associated gene expression changes are linked to AMD pathogenesis and highlight RPE lipid metabolism alteration

The expression of 3435 genes was found to be significantly associated with time in culture, either by fitting a linear or quadratic regression model. Enrichment analysis of these SCGs revealed alteration of pathways relevant to AMD pathogenesis like EMT, lipid metabolism, and calcium homeostasis that could contribute to drusen deposition and cell degeneration. Analysis of DEGs between 4 and 25 weeks in culture revealed genes whose expression over time may have a relationship not captured by the linear or quadratic regression models, additionally suggesting enrichment of oxidative stress during RPE degeneration. This large-scale temporal reprogramming of gene expression reflects progressive molecular changes in RPE cultures, providing insights into age-related dysfunction. Among lipid metabolism-related GO terms, the enrichment of processes such as “sphingolipid metabolic process,” “fatty acid metabolism,” and “lipid transport” highlights profound age-associated disruptions in lipid homeostasis. These findings align with the role of lipid dysregulation in AMD, where the accumulation of lipoprotein-like particles in Bruch’s membrane leads to lipid barrier formation, impaired nutrient and oxygen transport, hypoxia, and RPE detachment [10, 11]. Oxidative stress exacerbates these effects through lipid oxidation, inflammation, and immune activation [29], ultimately driving RPE and photoreceptor degeneration [30].

In fact, comparison of SCGs and DEGs found in RPE cell cultures with SCGs identified during in vivo ageing [17] or DEGs found between AMD and control subjects [18] revealed a higher similarity of transcriptomic patterns between the 6-month-old RPE cell model and RPE affected by AMD pathology. These results suggest that the transcriptomic changes observed in the RPE cell cultures more closely resemble pathological changes associated with AMD than natural ageing. While sub-epithelial deposit formation is characteristic of both ageing and early AMD, evidences collected in this cellular study and previous work on similar models support the idea that long-term cultivation of RPE cells can serve as drusen-on-a-dish models of early AMD [22].

### Temporal transcription changes are associated with lipid metabolism impairment during RPE degeneration

Significant alterations in genes associated with AMD-related molecular processes were observed during the transition from healthy ageing to early AMD in the simplified RPE cellular model. Particular attention was given to the significant alterations in lipid metabolism observed during ageing and AMD (Fig. 3). Angiopoietin like 4 (*ANGLPTL4*) gene, which encodes a signaling factor involved in lipid uptake, showed increased expression over time in our cell cultures. This upregulation could be linked to the breakdown of the RPE barrier. The *NPC2* gene, responsible for encoding a cholesterol transporter, exhibited a bell-shape trend with time in culture, resulting in a significant decrease in expression after 25 weeks of culture, consistent with the impairment observed in AMD. A deficiency in NPC2 has previously been associated with age-related maculopathies [31]. In contrast, the *TSPO* gene, which encodes another protein involved in cholesterol transport, was upregulated at the end of the experiment. Beyond cholesterol transport, TSPO plays a role in oxidative stress and inflammation, leading to its potential as target for antioxidant therapies in AMD [32].

Several genes involved in lipid metabolism, including *APOE*, *ABCA1*, and *ABCA7*, have been identified as risk factors for AMD [33, 34]. The APOE alleles epsilon 2 and epsilon 4 are associated with increased and decreased risk for AMD, respectively [35]. Similarly, the major variants of two polymorphisms in *ABCA1* are linked to an increased risk of the disease, whereas the minor variants are associated with decreased risk [36]. An AMD risk variant has also been described for *ABCA7* [37]. Although the genotype of our RPE cultures for these genes is unknown, their expression patterns are strikingly dysregulated, particularly the decrease in APOE expression found as the cultures age. *APOE* encodes a lipid transport protein found in drusen and implicated in several AMD-related pathogenic pathways, including interactions with the complement system and Aβ oligomerization. Additionally, its lipid transport function may contribute to the accumulation of cholesterol in drusen [38].

For *ABCA1* and *ABCA7*, we observed an increase in *ABCA1* expression and a decrease in *ABCA7* expression with time. ABCA1 protein mediates the efflux of cellular cholesterol and phospholipid to lipid-poor apolipoproteins. Impaired cholesterol efflux is thought to accelerate AMD progression by promoting formation of drusen and the deposition of extracellular lipids beneath the retina. Mice deficient in both ABCA1 and ATP binding cassette subfamily G member 1 (ABCG1)—another transporter involved in cholesterol efflux—exhibit drusenoid-like deposits, photoreceptor dysfunction, and impaired dark adaptation [39]. Notably, *ABCG1* was significantly downregulated at 25 weeks in our cultures (fold-change = 0.06, *q* value < 0.001). While ABCA1 primarily facilitates cholesterol efflux, ABCA7 specializes in the transfer of phospholipids, particularly sphingomyelin, and (lyso)phosphatidylcholine, to high-density lipoproteins (HDL), with limited involvement in cholesterol transport. However, a compensatory mechanism exists in which *ABCA7* can mediate cholesterol transfer in the absence or deficiency of *ABCA1*. Similarly, alterations in *ABCA7* can induce compensatory changes in *ABCA1* expression [40]. This mechanism aligns with the opposing expression trends of these genes observed in our cell cultures.

In the context of fatty acid metabolism, *FADS1* expression, which is implicated in the biosynthesis of polyunsaturated fatty acids (PUFAs) known to reduce the risk of AMD [41, 42], was found to be downregulated at 25 weeks. A polymorphism in *FADS1* has also been associated with AMD pathology. Additionally, the expression of genes in the *ELOVL* family showed significant downregulation, specifically *ELOVL2*, *ELOVL7*, and *ELOVL5* at the end of the 25-week culture period. Reduced *ELOVL2* expression, strongly correlated with ageing and methylation status, leads to a decrease in the synthesis of long-chain PUFAs and the accumulation of short-chain precursors in the endoplasmic reticulum, where ELOVL2 is localized. This disruption also results in mitochondrial dysfunction. Elongated PUFAs play a critical role in mitochondrial energy metabolism, particularly in the formation of bioactive lipids essential for mitochondrial function and energy production [43, 44]. Notably, *ELOVL2* knockout mice exhibit an accelerated ageing phenotype, increased inflammation, oxidative stress, and cellular senescence, with AMD-like alterations in the eye [45].

Emerging studies support the role of altered sphingolipid metabolism in AMD pathology [46], and variations in serum sphingolipid levels have been observed in AMD patients [33]. Ceramides (Cer), central intermediates in sphingolipid metabolism, are key regulators of inflammation, cell death, and inhibition of cellular proliferation. The involvement of ceramides in initiating AMD-related RPE cell death has been previously described [46]. In our RPE cultures, age-related dysregulation of genes involved in both the de novo and salvage pathways of ceramides synthesis was observed. Specifically, *SPTLC1*, *DEGS1*, and *CERS5* were upregulated, whereas *CERS1*, *CERS4*, and *CERS6* were downregulated. Similarly, the expression of *SMPD2* and *SMPD3*, which are involved in the synthesis of ceramides via sphingomyelin hydrolysis, decreased with ageing in culture. Sphingomyelin, like ceramide, acts as an inducer of apoptosis and inhibits cellular proliferation. The expression of *SGMS1*, a gene responsible for the synthesis of sphingomyelin from ceramides, was transiently upregulated at 12 and 17 weeks, but downregulated at the end of the 25 weeks in culture. Conversely, UGCG, which participates in the synthesis of glucosylceramides (GlcCer), was upregulated.

### Time in culture alters metabolites related to glutathione, purines, and membrane lipids

Time in culture significantly impacts the metabolome of RPE cells, leading to progressive and accumulative metabolic differences. PCA analysis (Fig. 4) revealed partial overlaps between the earliest time points, indicating that the metabolome of RPE cells cultured for 4 and 12 weeks share more similarities with each other than with cells cultured for 17 or 25 weeks. Heatmap and clustering analyses (Fig. 5 and Additional file 1: Fig. S3) using the most significantly altered metabolites (adjusted *p* value < 0.001 and fold-change > 16) identified three distinct patterns of abundance over time: (1) metabolites that decrease rapidly; (2) metabolites that decrease gradually; and (3) metabolites that increase over time.

#### Rapidly decreasing metabolites

Early-decreasing metabolites include S-glutathionyl-L-cysteine, a molecule involved in modulating signaling pathways in response to reactive oxygen species (ROS) and reactive nitrogen species (RNS) [47]. Its initial decline in abundance may indicate low oxidative stress levels at earlier time points, while its subsequent increase at 17 weeks correlates with increased oxidative stress. This is further bolstered up by the upregulation of the gene encoding the antioxidant enzyme SOD3. However, the decline of S-glutathionyl-L-cysteine levels between 17 and 25 weeks suggests either an effective oxidative stress response mediated by enzymes such as GPX and PRDX or advanced cellular degeneration. Similarly, adenosine levels decreased significantly, particularly after 17 weeks, suggesting that ageing affects the adenosinergic pathway in RPE cells. This disruption could be associated with chronic inflammation and other retinal alterations [48]. Lipid metabolism was also severely impacted, as evidenced by the rapid decline in multiple glycerophospholipids, including members of the phosphatidylethanolamines (PE), phosphatidylcholines (PC), and phosphatidylserines (PS) families. Lipids such as PC(20:5(5Z,8Z,11Z,14Z,17Z)/13:0), PS(15:0/20:5(5Z,8Z,11Z,14Z,17Z)), and PE(22:4(7Z,10Z,13Z,16Z)/13:0) showed a marked reduction over time. This decline in lipid metabolism is supported by both RNA and metabolite data, with implications for cellular signaling, autophagy, and inflammation, processes integral to ageing and AMD pathogenesis. These glycerophospholipids could potentially serve as biomarkers of maturation and degeneration [49].

#### Gradually decreasing metabolites

Metabolites in this group showed a slower decline and were predominantly reduced by the end of the experiment (25 weeks). Many of these metabolites are linked to pathways shared with early-decreasing compounds, reflecting the progression of metabolic dysfunction. Key metabolites include adenine, cyclic adenosine diphosphate ribose, inosine, 2-hydroxy-dAMP, and adenosine 2′,5′-bisphosphate, which are involved in the adenosinergic pathway. Their decay could have a progressive impact on energy metabolism, cell proliferation, survival, and extracellular signaling, contribution to the pathology of eye diseases [50]. Choline derivatives such as phosphorylcholine and succinylmonocholine also exhibited reduced levels at late time points. Phosphorylcholine, the main head group of several phospholipids, showed a decline consistent with the reduced glycerophospholipid biosynthesis observed after 12–17 weeks. Additionally, metabolites related to fatty acid metabolism, such as palmitoyl N-isopropylamide, decreased over time, further inhibiting lipid biosynthesis. Metabolites like 2-phenylacetamide, linked to phenylalanine metabolism, also declined. Interestingly, studies of AMD patients carrying the *HTRA1* risk allele (rs10490924) reported decreased systemic levels of 2-phenylacetamide compared to non-carriers, although the functional implications remain unclear [51].

#### Increasing metabolites

The third group of metabolites demonstrated an increase over time in culture, correlating with RPE ageing and degeneration. Lipid-related metabolites in this group include PE(O-20:0/17:2(9Z, 12Z)), the sphingolipid GlcCer(d16:1/18:0), and the choline ester (4-hydroxybenzoyl)choline. Among these, GlcCer(d16:1/18:0) showed the most significant upregulation over time, with an almost 18-logFC increase at 25 weeks compared to its abundance at 4 weeks. This dramatic increase aligns with the upregulation of *UGCG* expression at that stage (Fig. 3B), a key enzyme in GlcCer synthesis. Cer, the precursor of GlcCer, is known to regulate cell senescence, proliferation, inflammation, and apoptosis. The synthesis of GlcCer from Cer is often considered a protective mechanism to counteract the harmful effects of Cer accumulation, including its pro-apoptotic and autophagic actions [52]. GlcCer accumulation has been observed in conditions such as diabetic retinopathy, while alterations in sphingolipids, including Cer-mediated inflammation and autophagy, are associated with AMD [46]. Thus, the observed ceramide metabolism alterations in this study may serve as indicators of in vitro age-related RPE degeneration.

### Integrative transcriptomic and metabolomic analysis confirms alteration of glycerophospholipid and sphingolipid metabolism

The combined analysis of transcriptomic and metabolomic data confirms significant alterations in glycerolipid and glycerophospholipid metabolism at the end of the 25-week culture period (Fig. 6A). Other significantly enriched pathways include glutathione metabolism, lysine degradation, nitrogen metabolism, and sphingolipid metabolism. Visualization of these pathways provided a more comprehensive understanding of the biochemical changes in RPE cells, highlighting the enrichment of glycerophospholipid (Fig. 6B) and sphingolipid metabolism (Fig. 6C) at 25 weeks. While metabolomic or transcriptomic analyses alone have been widely used to study RPE ageing and AMD, the integration of these omic approaches remains relatively underexplored. Emri et al. employed transcriptomics to study RPE-specific gene changes, while Laíns et al. identified metabolomic alterations in AMD patients [48, 53]. The combination of transcriptomics and metabolomics, as presented here, provides a more comprehensive understanding of the underlying molecular changes.

#### Glycerophospholipid metabolism alteration

Most glycerophospholipids identified in RPE cultures belonged to the glycerophosphocholine (GPC), glycerophosphoethanolamine (GPE), and glycerophosphoserine (GPS) families (Fig. 6B). The primary biosynthetic pathway of PC is the CDP-choline pathway, which appeared inhibited at 25 weeks. Downregulation of key genes such as *PCYT1B* (encoding choline kinase) and *CHKA* (encoding CTP:phosphocholine cytidylyltransferase) was observed, along with decreased levels of PC metabolites. These findings indicate that the CDP-choline pathway is compromised early in time, with significant metabolic decline occurring between 17 and 25 weeks. Although PC species such as PC(22:4(7Z,10Z,13Z,16Z)/14:0) showed some recovery post-17 weeks, their levels remained below baseline, suggesting possible compensation via alternative pathways. Reduced CDP-choline levels at 25 weeks might elevate neurodegeneration risk, as CDP-choline has shown potential in inhibiting β-amyloid deposition in Alzheimer’s and glaucoma and improving neurobehavioral deficits in Parkinson’s disease by enhancing acetylcholine synthesis [54]. In vitro studies of ARPE19 cells have demonstrated that CDP-choline may prevent apoptosis by downregulating apoptosis-related genes and oxidative stress [55]. For PE species, declining levels corresponded with the downregulation of *ETNK2* (encoding ethanolamine kinase 2), implicating potential mitochondrial dysfunction and altered energy metabolism [56]. Similar metabolic disturbances, including those of PC/PE ratio, are observed in neurodegenerative diseases such as Alzheimer’s and Parkinson’s but have not been extensively studied in retinal degenerative conditions [57]. In this study, the PC/PE ratio progressively increased over time, with the most significant rise at 17 weeks, highlighting metabolic disruptions throughout the culture period [56].

#### Sphingolipid metabolism alteration

Enrichment of sphingolipid metabolism was characterized by a nearly 12-logFC increase in ceramide species Cer(d16:1/18:0) (Fig. 6C). Ceramide accumulation is a hallmark of several neurodegenerative diseases, including Alzheimer’s, Parkinson’s, and AMD. Ceramides are critical for cellular membrane structure and act as signaling molecules in cell proliferation, senescence, apoptosis, and autophagy [58]. In 25 weeks old RPE cultures, pathways involved in ceramide synthesis appeared to be inhibited, likely as a countermeasure to ceramide accumulation. Genes associated with de novo ceramide synthesis, including *CERS1*, *CERS4*, and *CERS6* (encoding ceramide synthase), were significantly downregulated, except for a slight upregulation of *CERS5*. Similarly, pathways producing ceramide via hydrolysis of ceramide phosphate (catalyzed by sphingosine-1-phosphate phosphatase, SGPP) and sphingomyelin (catalyzed by sphingomyelin phosphodiesterase, SMPD) were impaired. For instance, *SGPP2* was downregulated, while *GPP1* was upregulated, and two of the three *SMPD* genes (*SMPD2* and *SMPD3*) showed significant downregulation*.* Conversely, ceramide glycosylation was enhanced, as evidenced by the upregulation of *UGCG* and increased abundance of GlcCer(d16:1/18:0). Glucosylceramide accumulation may represent a protective mechanism against the cytotoxic effects of elevated ceramide, though its exact role in retinal health remains context-dependent. Ceramide accumulation has been linked to pro-inflammatory responses, ROS production, lysosomal dysfunction, and apoptosis, all of which contribute to RPE degeneration in AMD [59].

Altered lipid metabolism, particularly involving glycerophospholipids and sphingolipids, is believed to play a pivotal role in AMD onset [9]. Age-related thickening and stiffening of Bruch’s membrane—a structure separating the RPE from the choriocapillaris—impairs lipid transport and clearance, leading to cholesterol-rich deposit formation and drusen accumulation. This disrupts nutrient and oxygen cycling, contributing to RPE degeneration and photoreceptor loss, key events in AMD pathology [19, 20, 23, 60].

### Relationship between in vitro RPE degeneration and serum lipid profile alterations in AMD patients

The identification of altered lipid metabolites in RPE cells over time and their subsequent detection in the serum of AMD patients provides valuable insights into the systemic nature of lipid dysregulation in AMD pathology. A targeted serum analysis revealed detection of 18 out of 23 molecular compounds that exhibited significant changes during RPE deposit formation and in vitro deterioration. Most of these compounds were lipids, with the exception of two xenobiotics (HMMF and tris(butoxyethyl)phosphate) that showed no alteration between the serum of AMD patients and control subjects. Among the identified lipids, only four exhibited elevated serum levels in AMD patients, while 14 showed reduced levels. Notably, consistent correlations between changes in RPE cells and serum were observed for 12 out of 18 compounds. Four lipids displayed significant differences in AMD patients comparing to controls: GlcCer(d16:1/18:0) (1.23-fold increase, *p* < 0.001), PE(19:1(9Z)/22:2(13Z,16Z)) (0.34-fold decrease, *p* < 0.001), PE(15:0/20:3(5Z,8Z,11Z)) (0.66-fold decrease, *p* < 0.05), and PC(22:2(13Z,16Z)/13:0) (0.71-fold decrease, *p* < 0.05) (Fig. 7, Additional file 2; Table S5). These changes mirrored trends observed in RPE cultures over time, emphasizing the systemic impact of AMD on lipid metabolism. Interestingly, sphingolipid metabolism showed enrichment at the systemic level in AMD evidenced by increased serum levels of GlcCer(d16:1/18:0). Conversely, reductions in PE and PC in AMD patients confirmed impairments in glycerophospholipid metabolism.

Recent metabolomic studies have provided compelling evidence of altered lipid profiles in the serum of patients with AMD. Brown et al. reported altered levels of metabolites such as CySS, ω−3 PUFAs, glycerophospholipids, bile acids, and carnitines, highlighting potential disruptions in pathways related to cell membrane integrity, oxidative stress, and energy metabolism [61]. Laíns et al. identified decreased glycerophospholipid species in AMD serum by LC–MS/MS [48], while Semba et al. observed elevated LPC levels in neovascular AMD but not in geographic atrophy AMD [62]. Such lipidomic analyses consistently identify differences in glycerophospholipids, sphingolipids, and fatty acids, with a panel of 16 metabolites effectively distinguishing neovascular AMD cases from controls. These findings corroborate the lipid-rich composition of drusen, genetic associations with lipid homeostasis, and systemic metabolomic alterations in AMD [49].

Our findings emphasize the significance of lipid-related metabolites in AMD, particularly the dysregulation of the glycerophospholipid pathway, which is critical for maintaining cell membrane structure, signal transduction, and neural cell function [63]. In AMD, the reduced levels of PC suggest compromised cell membrane integrity, potentially stemming from altered lipid synthesis, transport, or degradation [64]. Given the phospholipid richness of photoreceptors and RPE cells, these changes likely impair the transduction of visual stimuli, contributing to retinal degeneration [65]. Furthermore, lipid imbalance may activate inflammatory pathways, exacerbating AMD disease progression [9]. The correlation between serum and RPE cell lipid alterations underscores the systemic nature of these changes and their potential as biomarkers. Monitoring lipidomic profiles in serum offers a non-invasive method for early detection, disease tracking, and predicting treatment outcomes in AMD patients. By quantifying lipid changes in RPE cultures and AMD patient serum, this study highlights the potential of lipidomic biomarkers for bridging laboratory research with clinical applications. Future research should focus on unraveling the functional roles of these lipids in AMD and exploring lipid-targeted therapeutic strategies. The integration of in vitro models with patient-derived samples provides a promising avenue for identifying actionable biomarkers and developing innovative interventions for managing this debilitating disease. Ultimately, these insights deepen our understanding of lipid dysregulation in AMD pathophysiology and reinforce the critical role of lipid homeostasis in disease prevention and treatment.

## Conclusions

This multiomic study of a primary RPE cell culture model of AMD provides valuable insights into the molecular mechanism underpinning early AMD pathogenesis. Over a 6-month period, RPE cells exhibited phenotypic deterioration, including loss of pigmentation, cell shape deformation, impaired antioxidant defenses, and compromised barrier integrity, reflecting changes associated with AMD risk. Combined metabolomic and transcriptomic analyses highlighted significant alterations in pathways related to lipid uptake, cholesterol transport, and glycerophospholipid and sphingolipid metabolism, emphasizing the critical role of lipid dysregulation in RPE dysfunction and AMD development. Key findings included time-dependent impairments in glycerophospholipid metabolism, ceramide accumulation, and mitochondrial dysfunction, with significant decreases in phospholipid species such as PC and PE. These changes mirror trends observed in serum lipid profiles of AMD patients, underscoring the systemic nature of AMD-related lipid dysregulation. Importantly, the consistent lipidomic alterations between RPE cultures and patient serum suggest a unified response, highlighting the translational potential of serum lipid profiling as a diagnostic tool and biomarker for monitoring disease progression.

Despite the advancements offered by our culture system, this study has several limitations. The imposed physical barrier in the RPE culture model, while effective for inducing AMD-like phenotypes, may not fully capture the complexity of in vivo processes. Additionally, while our findings underscore the importance of lipid metabolism in AMD, reversing these changes remains challenging due to the multifactorial and heterogeneous nature of the disease. Future experiments could also investigate whether normalizing lipid metabolism in vitro restores cellular function or mitigates AMD-like phenotypes. These efforts could provide critical insights into the functional role of lipid alterations in AMD and their potential reversibility.

## Methods

### RPE cellular model of AMD

Primary RPE cell cultures were established with human fetal RPE cells acquired from ScienCell (Cat. No. 6540) derived from a single donor, seeded at passage 3 into 12-well Transwell® inserts (Corning Inc., Cat. No. CLSS3460) pre-coated with 2% v/v Geltrex® matrix (Thermo Fisher Scientific; Cat. No. A1413202). Cultures were maintained for up to 6 months (25 weeks) following protocols established in prior studies [15, 19]. Samples for protein immunolocalization, transcriptomic, and metabolomic analyses were collected from independent cell cultures at specified time points (4, 12, 17, and 25), with three biological replicates per analysis and time point.

### Characterization of RPE cell cultures

Throughout the study, cell cultures were monitored by optical microscopy using a Leica DM II LED microscope (Leica Microsystems) to evaluate phenotypic characteristics. TEER was measured using a Millicell ERS-2 Voltohmmeter (Merck Millipore, ref. MERS00002) during medium changes to assess epithelial integrity. Immunocytochemistry was performed to detect specific RPE proteins, including ZO-1, CLDN19, BEST1, and APOE, along with HAP deposits, at all time points (see Additional file 1 for protocol details). Imaging was conducted using a Leica TCS-SP8X confocal fluorescence microscope (Leica Microsystems).

### Transcriptomic analysis

RNA extraction was performed using the RNeasy Mini Kit (Qiagen) following the manufacturer’s instructions (Additional file 1). RNA quality control, library construction, RNA sequencing, and quantitative analysis were conducted by BGI Genomics (Beijing Genomics Institute, Shenzhen, China) using the BGISEQ-500 platform. Two complementary statistical analyses were conducted to identify genes whose expression was associated with time in culture: regression analysis, in which time was considered a continuous variable to define SCGs, and differential expression analysis, in which time was considered a discrete variable to define DEGs.

For regression analysis, counts were normalized using DESeq2 [66] and linear and quadratic models were implemented using lm function of R (v4.4.2). SCGs were defined as genes whose expression over time was described by a regression model with global statistical significance and for each term of the regression (*p* value adjusted with Benjamini–Hochberg method < 0.05). For SCGs that passed the significance cutoff with both a linear and quadratic regression model, ANOVA test was additionally conducted to determine if the quadratic term was statistically relevant for data variance (*p* value < 0.05). As for differential expression analysis, DEGs were identified using Dr. Tom Data Visualisation Solution (http://biosys.bgi.com. Accessed 26 January 2024) and DESeq2 method [66], defining statistical significance as adjusted *p* value (*Q* value) ≤ 0.05. DEGs and SCGs were mapped to GO database and enriched biological processes were identified as those having *Q* value ≤ 0.05, using Dr. Tom and ShinyGO 0.81, respectively.

A comparison with datasets containing real sample data was carried out to validate SCGs and DEGs found in RPE cell cultures. Dataset “Ageing” was provided by Butler et al. [17] and accessed through Gene Expression Omnibus repository (accession code: GSE159435). SCGs identified by authors through linear regression modeling were selected, while genes fitting a quadratic regression model were added by implementing a mixed model considering “Batch” and “Sex” as covariates to DESeq2-normalized data using R [67]. Dataset “AMD” was provided by Orozco et al. [18] and accessed through Zenodo repository (accession code: 10.5281/zenodo.7020215), selecting pre-existing treated data. Comparison of SCGs and DEGs in RPE cell cultures with “Ageing” and “AMD” datasets relied on different data visualization approaches generated with R packages based on ggplot2.

### Untargeted metabolomic analysis

#### Metabolite quenching and extraction

Cell metabolites were quenched and extracted with cold MeOH:H_2_O (80:20, − 20 °C) containing isotopically enriched internal standards. Quality control (QC) samples were prepared by pooling equal volumes from each biological replicate and analyzed after every fifth sample to monitor LC–MS performance, stability, and reproducibility.

#### LC–MS and LC–MS/MS analysis of cell extracts

Samples were analyzed using an Agilent 1290 Infinity II LC system coupled to an Agilent 6560B ion mobility quadrupole-time-of-flight mass spectrometer (IM-QTOF-MS) equipped with dual JetStream ESI source (Agilent G1607A) and the MassHunter WorkStation 11.0. A reference solution containing purine and hexakis(1H,1H,3H-tetrafluoropropoxy)phosphazene for mass correction was added using the second ESI source. Chromatographic conditions, ion source settings, and MS parameters were optimized using QCs, with detailed parameters available in Additional file 1. Data acquisition was performed in both positive and negative ionization modes and samples were analyzed in randomized order. Targeted MS/MS analysis was carried out to obtain product ions of metabolites of interest with statistically significant differences.

#### Raw data processing and statistical analysis

Raw data were processed using Profinder B10.0 software (Agilent) and refined data were exported as CEF files. Statistical analyses were performed with MPP software (Agilent, v15.1), after normalizing the abundance of each feature to the sum of entities present across all samples (see Additional file 1), and with cell weight extract used as external scalar, using Kruskal–Wallis test, with Benjamini–Hochberg correction for multiple comparisons.

### Integrative analysis

Joint pathway analysis combining transcriptomic and metabolomic data was performed using Metaboanalyst 5.0 (https://metaboanalyst.ca/. Accessed 5 May 2024), Pathview (https://pathview.uncc.edu/. Accessed 8 June 2024), and KEGG pathway database [68]. Pathway significance was calculated using overall *p* value combination method, and impact was estimated through degree centrality topology measurement.

### Targeted relative quantification of lipids in AMD patients and control subjects

#### Study subjects

An observational, prospective, case-controlled study was conducted with 40 participants: 20 patients diagnosed with dry AMD and 20 healthy controls. The study adhered to the Declaration of Helsinki principles for Biomedical Research Involving Human Subjects and was approved by the Clinical Research Ethics Committee of the Principality of Asturias (Oviedo, Spain). Participants were recruited at the Fernández-Vega Institute of Ophthalmology (Oviedo, Spain) based on inclusion and exclusion criteria, with demographic details provided in Additional file 2: Table S4 [69]. Written informed consent was obtained from all participants.

#### Serum sampling and preparation

Peripheral blood was collected into 5-mL Z Serum Sep Clot Activator tubes (Vacuette, Madrid, Spain). After centrifugation (1800 g for 18 min at 4 °C), serum was stored at − 80 °C. Serum metabolites were extracted by diluting 10 µL of serum in 100 µL of cold methanol:methyl tert-butyl ether (MTBE, 1:1) with 10 mM of ammonium formate, containing deuterated lipid standards (Avanti Research, USA) [0.0363 ppm of 18:1 (d7) MG; 0.4545 ppm of 18:1(d7) LPC; 0.5454 ppm of 18:1 (d9) SM; 2.9090 ppm of 15:0–18:1(d7) PC; 0.1820 ppm of 15:0–18:1(d7) DG; 1.00 ppm of 15:0–18:1 (d7)−15:0 TG; 0.0901 ppm of 15:0–18:1(d7) PE; 0.0901 ppm of 15:0–18:1(d7) PS; 9.6364 ppm of 15:0–18:1(d7) PG; 0.1819 ppm of 15:0–18:1(d7) PI; 0.0910 ppm of 18:1(d7) Lyso PE; 6.3636 ppm of 18:1(d7) Chol Ester]. Samples were vortexed, sonicated (1 h), and centrifuged (13,000 g for 10 min), with the supernatant analyzed by LC–MS. QC samples were prepared by pooling equal volumes from all serum extracts.

#### Relative quantification of RPE molecules altered with time by LC–MS

Serum lipids were analyzed using a targeted approach on an Acquity UPLC CSH C18 column (Waters) with optimized chromatographic and MS conditions (see Additional file 1). Relative quantitative analysis was performed by normalizing lipid levels to internal standards. Statistical analyses were conducted using either the Mann–Whitney test or unpaired *t*-test, depending on data normality assessed by the Kolmogorov–Smirnov test. Adjusted *p* values were calculated using the Benjamini–Hochberg method to account for multiple comparisons.

## Supplementary Information

Additional file 1: Supporting information. Table S1 Enriched biological processes with time in culture. SCGs identified in RPE cell cultures through linear or quadratic regression models were compared to genes annotated to GO biological processes database using ShinyGO 0.81. Statistical significance was monitored as false discovery rate (FDR) and adjusted for multiple comparisons through Benjamini–Hochberg method. The analyses included data from three biological replicates of cell cultures at 4, 12, and 17 weeks, as well as two additional replicates at 25 weeks. Fig. S1 Enrichment of biological processes according to GO database between 4, 12, 17, and 25 weeks’ comparisons. Bubble chart of the 20 most significantly enriched biological processes when comparing (A) 4 weeks (*n* = 3) vs 12 weeks (*n* = 3), (B) 4 weeks (*n* = 3) vs 17 weeks (*n* = 3), (C) 12 weeks (*n* = 3) vs 17 weeks (*n* = 3), (D) 12 weeks (*n* = 3) vs 25 weeks (*n* = 2), and (E) 17 weeks (*n* = 3) vs 25 weeks (*n* = 2). Each process is represented by a bubble and indicated in the Y-axis. The size of the bubble shows the number of DEGs annotated to that GO term and its color, the significance of its enrichment (*q* value). X-axis represents the enrichment ratio of each process (ratio between the number of DEGs annotated to the GO term and the total number of genes annotated to that GO term). Fig. S2 Expression of RPE-specific genes over time. (A) DEGs identified between 4 (*n* = 3), 12 (*n* = 3), 17 (*n* = 3), and 25 (*n* = 2) time points. Fold-changes and statistical significance were obtained by DESeq2 method. ns: *q* value > 0.05; *: *q* value < 0.05; **: *q* value < 0.01; ***: *q* value < 0.001. (B) SCGs identified through linear and quadratic regression models. Equation of the regression curve is shown together with the associated coefficient of determination (*R*^2^) and *p* value adjusted by Benjamini–Hochberg method. Fig. S3 Heatmap of hierarchical clustering analysis of differential molecular features in 25-week-old (*n* = 3 biological replicates, each measured 3 times) RPE cells versus 4- (*n* = 3 biological replicates, each measured 3 times), 12- (*n* = 3 biological replicates, each measured 3 times), and 17-week-old (*n* = 3 biological replicates, each measured 3 times) cells, (A) in positive ionization mode and, (B) in negative ionization mode. Significantly different compounds were identified using the Kruskal–Wallis test with Benjamini–Hochberg correction (adjusted *p* value < 0.05 and fold-change > 2).

Additional file 2: Tables S2–S5. Table S2 Small molecules detected and identified in positive ionization mode. A total of 185 compounds where tentatively identified using ID browser, showing the normalized averaged data for each of the tentatively identified compounds. Table S3 Small molecules detected and identified in negative ionization mode. A total of 68 compounds where tentatively identified using ID browser, showing the normalized averaged data for each of the tentatively identified compounds. Table S4 Demographic characteristics of AMD patients (*n* = 20) and control subjects (*n* = 20). Table S5 Comparison of selected lipids levels in serum samples from control (*n* = 20) and AMD patients (*n* = 20). Lipid levels were normalized to the sample volume and expressed as the mean ± standard deviation across 20 biological replicates for each group. Group comparisons were conducted using the Mann–Whitney or unpaired *t*-test, depending on data distribution normality assessed via the Kolmogorov–Smirnov test. To account for multiple comparisons, *p* values were adjusted using the Benjamini–Hochberg method. Statistical significance is indicated as follows: ns: adjusted *p* value > 0.05; *: adjusted *p* value < 0.05; **: adjusted *p* value < 0.01; ***: adjusted *p* value < 0.001.

Additional file 3: Individual data values of gene expression over time (4, *n* = 3; 12, *n* = 3; 17, *n* = 3; and 25 weeks, *n* = 2).

Additional file 4: Individual data values for metabolomics over time (4, 12, 17, and 25 weeks, *n* = 3 biological replicates of each time point, each measured 3 times), in positive and negative ion mode.

## Data Availability

All data generated or analyzed during this study are included in this published article, its supplementary information files and publicly available repositories. The metabolomics datasets have been converted to mzML format and uploaded to Zenodo (https://zenodo.org/records/14266687 and 10.5281/zenodo.14266687). RNA sequencing data have been deposited in the ArrayExpress database under accession number E-MTAB-14705 (https://www.ebi.ac.uk/biostudies/arrayexpress/studies/E-MTAB-14705). Individual data values from transcriptomics are included in Additional File 3. Individual data values from metabolomics are included in Additional File 4.

## Abbreviations

*ABCA1*: ATP binding cassette subfamily A member 1
ABCA7: ATP binding cassette subfamily A member 7
ABCG1: ATP binding cassette subfamily G member 1
AMD: Age-related macular degeneration
*ANGPTL4*: Angiopoietin-like 4
APOE: Apolipoprotein E
BEST1: Bestrophin 1
Cer: Ceramides
*CERS1*: Ceramide synthase 1
*CERS4*: Ceramide synthase 4
*CERS5*: Ceramide synthase 5
*CERS6*: Ceramide synthase 6
*CHKA*: CTP:phosphocholine cytidylyltransferase
CLDN19: Claudin 19
*DCT*: Dopachrome tautomerase
DEGs: Differentially expressed genes
*DEGS1*: Delta 4-desaturase sphingolipid 1
*ELOVL*: Fatty acid elongases
EMT: Epithelial-to-mesenchymal transition
*ETNK2*: Ethanolamine kinase 2
FADS1: Fatty acid desaturase 1
GlcCer: Glucosylceramides
GP: Gene Ontology
GPC: Glycerophosphocholine
GPE: Glycerophosphoethanolamine
GPS: Glycerophosphoserine
HAP: Hydroxyapatite
HDL: High-density lipoproteins
IM-QTOF-MS: Ion mobility quadrupole-time-of-flight mass spectrometer
LC-MS: Liquid chromatography-mass spectrometry
*LRAT*: Lecithin retinol acetyltransferase
*MITF*: Melanocyte inducing transcription factor
MPP: Mass Profiler Professional
*NPC2*: Niemann-Pick disease intracellular cholesterol transporter 2
PC: Phosphatidylcholines
PCA: Principal component analysis
*PCYT1B*: Phosphate cytidylyltransferase 1B, choline
PE: Phosphatidylethanolamines
*PLTP*: Phospholipid transfer protein
PS: Phosphatidylserines
PUFAs: Polyunsaturated fatty acids
QC: Quality control
*RDH5*: Retinol dehydrogenase 5
*RGR*: RPE-retinal G-coupled receptor
*RLBP1*: Retinaldehyde binding protein
RNS: Reactive nitrogen species
ROS: Reactive oxygen species
RPE: Retinal pigment epithelium
*RPE65*: Retinal pigment epithelium-specific 65 kDa protein
SCGs: Significantly correlated genes
SGPP: Sphingosine-1-phosphate phosphatase
SM: Sphingomyelin
*SMPD2*: Sphingomyelinase 2
*SMPD3*: Sphingomyelinase 3
*SPTLC1*: Serine palmitoyltransferase long chain base subunit 1
TEER: Transepithelial electrical resistance
*TSPO*: Translocator protein
*TYR*: Tyrosinase
*TYRP1*: Tyrosinase-related protein 1
*UGCG*: UDP-glucose ceramide glucosyltransferase
ZO-1: Zonula occludens-1
