# Supplementary material for: Dysregulated lipid metabolism in a retinal pigment epithelial cell model and serum of patients with age-related macular degeneration
Source: BMC Biol. 2025 Apr 12;23:96. doi: 10.1186/s12915-025-02198-8 (PMC11993946; doi:10.1186/s12915-025-02198-8)
Supplement: Supplementary file 1 — Additional file 1: Supporting information. Table S1 Enriched biological processes with time in culture. SCGs identified in RPE cell cultures through linear or quadratic regression models were compared to genes annotated to GO biological processes database using ShinyGO 0.81. Statistical significance was monitored as false discovery rate (FDR) and adjusted for multiple comparisons through Benjamini–Hochberg method. The analyses included data from three biological replicates of cell cultures at 4, 12, and 17 weeks, as well as two additional replicates at 25 weeks. Fig. S1 Enrichment of biological processes according to GO database between 4, 12, 17, and 25 weeks’ comparisons. Bubble chart of the 20 most significantly enriched biological processes when comparing (A) 4 weeks (n = 3) vs 12 weeks (n = 3), (B) 4 weeks (n = 3) vs 17 weeks (n = 3), (C) 12 weeks (n = 3) vs 17 weeks (n = 3), (D) 12 weeks (n = 3) vs 25 weeks (n = 2), and (E) 17 weeks (n = 3) vs 25 weeks (n = 2). Each process is represented by a bubble and indicated in the Y-axis. The size of the bubble shows the number of DEGs annotated to that GO term and its color, the significance of its enrichment (q value). X-axis represents the enrichment ratio of each process (ratio between the number of DEGs annotated to the GO term and the total number of genes annotated to that GO term). Fig. S2 Expression of RPE-specific genes over time. (A) DEGs identified between 4 (n = 3), 12 (n = 3), 17 (n = 3), and 25 (n = 2) time points. Fold-changes and statistical significance were obtained by DESeq2 method. ns: q value > 0.05; *: q value < 0.05; **: q value < 0.01; ***: q value < 0.001. (B) SCGs identified through linear and quadratic regression models. Equation of the regression curve is shown together with the associated coefficient of determination (R2) and p value adjusted by Benjamini–Hochberg method. Fig. S3 Heatmap of hierarchical clustering analysis of differential molecular features in 25-week-old (n = 3 biological [file 12915_2025_2198_MOESM1_ESM.docx]

**SUPPORTING INFORMATION**

**Dysregulated lipid metabolism in a retinal pigment epithelial cell model and serum of patients with age-related macular degeneration**

**Ana Álvarez-Barrios^1,2^, Lydia Álvarez^1,*^, Pilar Sáenz de Santa María^1,3^, Montserrat García^3^, Jorge R. Álvarez-Buylla^4^, Rosario Pereiro^2^, Héctor González-Iglesias^4,*^**

^1^Fundación de Investigación Oftalmológica, Oviedo, Spain

^2^Department of Physical and Analytical Chemistry, University of Oviedo, Oviedo, Spain

^3^Instituto Oftalmológico Fernández-Vega, Oviedo, Spain

^4^Instituto de Productos Lácteos de Asturias, Consejo Superior de Investigaciones Científicas (IPLA-CSIC), Oviedo, Spain

***CORRESPONDENCE:** Lydia Álvarez ([l.alvarez@fio.as](mailto:l.alvarez@fio.as)) and Héctor González-Iglesias ([hectorgi@ipla.csic.es](mailto:hectorgi@ipla.csic.es))

**METHODS**

**Characterization of RPE cell cultures by immunocytochemistry**

Specific RPE cell proteins, including zonula occludens-1 (ZO-1), claudin 19 (CLDN19), bestrophin 1 (BEST1) and apolipoprotein E (APOE), were detected using immunocytochemistry at 4, 12, 17 and 25 weeks in culture. Cell were fixed with 4% (*v/v)* paraformaldehyde (methanol-free) for 15 min and stored in PBS at 4ºC before immunostaining. Fixed cell were transferred to microscope slides by retrieving the polyester microporous membrane from transwell inserts with tweezers. Immunocytochemistry involved permeabilization, blocking, and incubation with primary and secondary antibodies. The following primary antibodies and concentrations were used: Anti-ZO1 antibody (1:100; Thermo Fisher Sci., Ref. 617300), anti-CLDN19 antibody (1:100; NOVUS, Ref. H00149461-M02), anti-BEST1 antibody (1:50; Sigma-Aldrich, Ref. MAB5466), and anti-APOE antibody (1:100; Sigma-Aldrich, Ref. AB947). Secondary antibodies conjugated to AF594 or AF488 fluorophores were used. Hydroxyapatite (HAP) deposits were visualized using the OsteoSense^TM^ 680EX probe (PerkinElmer, Ref. NEV10020EX) at a concentration of 20 μM for 20 min at room temperature before mounting and imaging.

**RNA extraction**

Cultures were washed twice with PBS and lysed directly on the transwell inserts using RNeasy Mini Kit (Qiagen). Lysates were collected into RNase-free Eppendorf tubes, and RNA isolation was conducted following the instructions of the manufacturer.

**Untargeted metabolomics**

***Metabolite quenching and extraction***

Metabolite extraction was conducted on dry ice to prevent degradation. Samples were washed with PBS, and 100 μL MeOH:H_2_O (80:20, -20ºC) were added to quench the metabolism. Cells were detached using a scraper, collected into Eppendorf tubes, and completed with additional volume of MeOH:H_2_O (80:20, -20ºC) to recover all remnant cells. Suspensions were sonicated in an ice-water bath for 5 min to lysate the cells, centrifuged at 16,000 g at 4ºC for 20 min, and the supernatant was filtered (0.22 μm) before storage at -80ºC. The extracting solution MeOH:H_2_O (80:20) contained a mixture of isotopically enriched low molecular mass standards to evaluate extraction efficiency and instrumental performance: 500 ppb of glycoursodeoxycholic acid (C_26_H_39_D_4_NO_5_), from Sigma Aldrich; and 40 ppb of L-tryptophan (^13^C_11_H_12_N_2_O_2_), L-Leucine (^13^C_6_H_7_NO_2_), L-alanine (^13^C_3_H_7_NO_2_), L-tyrosine (^13^C_6_C_3_O_3_NH_11_), L Phenylalanine [^13^C_6_C_3_O_2_NH_11_), D-glucose (^13^C_6_O_6_H_12_), caffeine (^13^C_3_C_5_O_2_N_4_H_10_), D-sucrose (^13^C_6_C_6_O_11_H_22_), stearic acid (^13^C_18_O_2_NaH_35_), benzoic acid (^13^C_6_CO_2_H_6_), sodium octanoate (^13^C_18_O_2_NaH_35_), sodium propionate (^13^C_3_NaO_2_H_5_), sodium succinate (^13^C_4_Na_2_H_4_O_4_), sodium citrate (^13^C_3_C_3_O_7_Na_3_H_5_), sodium stearate (^13^C_18_O_2_NaH_35_) from Cambridge Isotope Laboratories.

***LC-MS and LC-MS/MS analysis of cell extracts***

Samples were analyzed using an LC system was fitted with a reverse phase column Zorbax RRHD Extend-C18 (80Å, 2.1 x 150 mm, 1.8 µm, Agilent Technologies). Two mobile phases were used to create a polarity gradient: water (phase A) and methanol (phase B), each with ionization-aiding additives (0.1% v/v formic acid for positive ion mode, and 0.1% v/v ammonium formate for negative ion mode). Mobile phase flow rate was 0.3 mL min^-1^ and temperature system 40ºC. For positive ion mode, the selected gradient consisted of 5% B for 0.5 min; 5 to 100% B over 0.5 to 8 min; 100% for 8 to 15 min; 100 to 5% B over 15 to 16 min. For negative ion mode, the selected gradient consisted of 5% B for 0.5 min; 5 to 100% B over 0.5 to 8 min; 100% for 8 to 15 min; 100 to 5% B over 15 to 16 min. Injected volume of samples, QC and blank solutions was 2 μL and all of them were run in triplicates.

MS parameters: Nitrogen was used as drying gas at a temperature of 250 ºC, a sheath gas temperature of 350 ºC and a sheath gas flow rate of 13 L/min. The nebulizer gas pressure was 30 psi, the ESI capillary voltage was 3500 V, the nozzle voltage 0 V, the fragmentor was set to 400 V and the octapole RF Vpp was 750 V. Acquisition was carried out in 2 GHz high resolution mode and mass range of 50-1000 m/z, with 2 spectra/s rate, 500 ms/spectrum and data storage in centroid. Targeted MS/MS analysis were carried out to obtain product ions of metabolites. Selected precursor ions were fragmented using 10, 20 and 40 eV and the scan time 0.9 spectra/s in MS and 3 spectra/s in MS/MS.

***Raw data processing and statistical analysis***

Raw data were processed using Profinder B10.00 software (Agilent) for chromatogram alignment, noise peaks reduction, and QC stability checks. Recursive Feature Extraction (RFE) was applied using Find by Ion algorithm with retention time and m/z tolerance of ± 0.15 min and 5 ppm. Features were filtered for a minimum absolute height of 10,000 counts and a minimum confidence score of 80 in at least 80% of files in at least one sample group. Statistical analyses were performed with Mass Profiler Professional (MPP) software (Agilent, version 15.1). The data were normalized by an external scalar using cell weight extract and by normalizing the abundance of each feature to the total sum of intensities from all features present in the samples. Normalization strategy was confirmed using glutamic acid analysis, showing constant values with no significant differences across all conditions studied.

Unsupervised principal component analysis (PCA) was performed with mean centring and scaling to assess the intra- and inter-group variance. Normality was tested through Shapiro Wilk. Since most of the obtained features didn’t pass the normality test, Kruskal-Wallis with Benjamini-Hochberg correction was chosen to compare the mean of each feature between groups. Compounds with a p-value < 0.05 and fold-change > 2 were considered significantly different. Tentative identification of statistically significant metabolites was conducted using the IDBrowser function of MPP by matching the obtained features with metabolites in the METLIN database based on accurate mass (mass accuracy window of 5 ppm), isotopic pattern and retention time. Molecular Formula Generator algorithm was used to compare the molecular formula generated by the software and the proposed compound from the database. The identities of altered metabolites were further confirmed by MS/MS analysis.

**Targeted lipid analysis in the serum of AMD patients and control subjects**

***Study Population***

Both AMD and control groups underwent comprehensive ophthalmic evaluations, including slit-lamp biomicroscopy and funduscopy of both eyes. Additional diagnostic procedures for AMD patients included fluorescein fundus angiography or optical coherence tomography. Eligibility for the AMD group required evidence of geographic atrophy in at least one eye, without signs of choroidal neovascularization. Control subjects, aged ≥ 65, were free of signs of AMD or glaucoma, specifically any drusen, including reticular pseudodrusen, and exhibited no pigmentary abnormalities in either eye, corresponding to stage 1 of the Clinical Age-Related Maculopathy Staging classification. Exclusion criteria for both AMD patients and controls included any other significant ocular pathologies, such as diabetic retinopathy, ocular surface diseases (including dry eye syndrome), or glaucoma. Participants with a history of ocular surgery other than cataract extraction, as well as those using topical eye medications or contact lenses, were excluded.

***LC-MS analysis of serum***

Chromatographic separation of extracted serum samples (1 µL) was performed using an Acquity UPLC CSH C18 column (130 Å 1.7 µm, 2.1 mmx100 mm, Waters) at a temperature of 60ºC with a flow rate of 0.3 mL/min. Optimized mobile phases consisted of 10 mM ammonium formate in acetonitrile:water (60:40) as phase A, and 10 mM ammonium formate isopropanol:acetonitrile (90:10) as phase B. The chromatographic gradient was as follows: 50 % B for 0.5 min; 53 % B from 0.5 to 4 min; 55 % B from 4 to 7 min; 65 % B from 7 to 12 min; 80 % B over 12 to 14 min; 99 % B from 14 to 20 min; 99 %B for 1 min; 50 % B over 2 to 22 min. The column was re- equilibrated for 3 min using the initial solvent composition.

MS instrumental parameters included N_2_ drying gas at 250 °C and sheath gas temperature at 300 °C with flow rate of 11 L/min. Nebulizer gas pressure was set to 35 psi. Instruments settings included a fragmentor voltage of 160V, an octapole RF voltage of 750 V, and acquisition performed in 2 GHz high-resolution mode. The mass range was set between 100–1700 m/z, with a spectra acquisition rate of 2 spectra/s and an acquisition time of 500 ms/spectrum. Data were stored in centroid, in both positive and negative ion modes. For targeted MS/MS analysis, the mass range was 100-1700 m/z, with spectra rate of 3 spectra/s (333.3 ms/spectrum). Precursor ion selection was performed with an isolation 1.3 amu, using collision energy of 20 eV. A maximum of three precursors ions per cycle was analyzed, with precursor abundance determining scan speed of 25000 counts/spectrum. A minimum threshold of 5000 counts was set for MS/MS analysis, and precursor ions were sorted by priority, with rank 1, 2, or unknown ions receiving preference.

The MS analysis was performed using a targeted approach, operating the instrument in both positive and negative ionization modes. A PCDL (Personal Compound Database and Library), containing accurate mass with retention time and one or more MS/MS curate mass spectra was created from the one and third group of molecules consisting of compounds decreasing with time at a quick rate and compounds increasing with time, respectively, namely PC(22:6(4Z,7Z,10Z,13Z,16Z,19Z)/14:0), PE(19:1(9Z)/22:2(13Z,16Z)), PE(15:0/20:3(5Z,8Z,11Z)), PE(P-18:1(9Z)/20:4(6E,8Z,11Z,14Z)(5OH[S])), PE(15:0/16:0)[U], HMMF, PS(22:4(7Z,10Z,13Z,16Z)/15:1(9Z)), PC(22:2(13Z,16Z)/13:0), PC(20:5(5Z,8Z,11Z,14Z,17Z)/13:0), PE(20:2(5Z,8Z)/18:0)[S], PS(15:0/20:5(5Z,8Z,11Z,14Z,17Z)), Narasin, PE(22:4(7Z,10Z,13Z,16Z)/13:0), (25R)-3beta-hydroxy-7-oxo-5-cholestenoic acid, PE(22:6(4Z,7Z,10Z,13Z,16Z,19Z)/16:1(9Z)), PE(22:6(4Z,7Z,10Z,13Z,16Z,19Z)/19:1(9Z)), Roxatidine acetate, S-Glutathionyl-L-cysteine, GlcCer(d16:1/18:0), 1-(6-[3]-ladderane-hexanoyl)-2-(8-[3]-ladderane-octanyl)-sn-glycerophosphocholine, PE(O-20:0/17:2(9Z,12Z)), Cer(d16:1/18:0), and Tris(butoxyethyl)phosphate. The identification for each compound were based on its molecular formula, exact mass, MS/MS spectra, and retention time by batch targeted feature extraction using the Profinder v10 software (Agilent). The abundance of each compound was used for their relative quantification by proper compound normalization against deuterated lipid internal standards of known concentration, applying the criterion that they belong to the same family or have similar retention times. Lipid levels were normalized to the sample volume and presented as the mean ± standard deviation of 20 biological replicates. Statistical comparisons between groups were conducted using either the Mann-Whitney test or unpaired t-test, depending on data normality, which was assessed using the Kolmogorov-Smirnov test. P-values were adjusted for multiple comparisons using the Benjamini-Hochberg method.

**RESULTS**

**Regression analysis and pathway enrichment to identify time-associated genes in culture**

Regression analysis of biological processes based on SCGs identified 1,000 significantly altered GO terms. Three major groups of GO terms were identified, associated with lipid metabolism, epithelial-to-mesenchymal transition (EMT), and calcium deposition (Table S1).

**Table S1. Enriched biological processes with time in culture.** SCGs identified in RPE cell cultures through linear or quadratic regression models were compared to genes annotated to GO biological processes database using ShinyGO 0.82 (<https://bioinformatics.sdstate.edu/go/>. Accessed 28 December 2024). Statistical significance was monitored as False Discovery Rate (FDR) and adjusted for multiple comparisons through Benjamini-Hochberg method. Analyses included data from 3 biological replicates of the cell cultures of 4, 12, and 25 weeks and 2 replicates of 25 weeks.

| **GO term** | **Description** | **Gene number** | **Pathway Gene** | **Enrichment FDR** |
| --- | --- | --- | --- | --- |
| **Lipid metabolism** | | | | |
| GO:0006643 | Membrane lipid metabolic process | 69 | 226 | 2.00E-06 |
| GO:0006687 | Glycosphingolipid metabolic process | 31 | 73 | 4.58E-06 |
| GO:0006665 | Sphingolipid metabolic process | 57 | 179 | 5.59E-06 |
| GO:0030149 | Sphingolipid catabolic process | 21 | 40 | 6.82E-06 |
| GO:0006664 | Glycolipid metabolic process | 42 | 117 | 6.87E-06 |
| GO:1903509 | Liposaccharide metabolic process | 42 | 118 | 8.65E-06 |
| GO:0046466 | Membrane lipid catabolic process | 22 | 44 | 9.82E-06 |
| GO:0006629 | Lipid metabolic process | 308 | 1516 | 5.33E-05 |
| GO:0001573 | Ganglioside metabolic process | 19 | 38 | 5.37E-05 |
| GO:0046514 | Ceramide catabolic process | 16 | 29 | 6.69E-05 |
| GO:0044255 | Cellular lipid metabolic process | 231 | 1103 | 0.000116952 |
| GO:0044281 | Small molecule metabolic process | 374 | 1936 | 0.000377422 |
| GO:0061526 | Acetylcholine secretion | 8 | 10 | 0.000456929 |
| GO:0006689 | Ganglioside catabolic process | 10 | 15 | 0.000465803 |
| GO:0015871 | Choline transport | 10 | 15 | 0.000465803 |
| GO:0006672 | Ceramide metabolic process | 37 | 119 | 0.000729413 |
| GO:0008291 | Acetylcholine metabolic process | 9 | 13 | 0.000732533 |
| GO:0046479 | Glycosphingolipid catabolic process | 12 | 22 | 0.000974208 |
| GO:0015870 | Acetylcholine transport | 8 | 11 | 0.001143837 |
| GO:0008610 | Lipid biosynthetic process | 164 | 776 | 0.001219173 |
| GO:0006644 | Phospholipid metabolic process | 101 | 439 | 0.001301399 |
| GO:0019377 | Glycolipid catabolic process | 12 | 23 | 0.001536026 |
| GO:0008292 | Acetylcholine biosynthetic process | 7 | 9 | 0.001696341 |
| GO:0046467 | Membrane lipid biosynthetic process | 44 | 157 | 0.001822996 |
| GO:0008654 | Phospholipid biosynthetic process | 70 | 293 | 0.004266648 |
| GO:0030148 | Sphingolipid biosynthetic process | 34 | 117 | 0.004458583 |
| GO:0033993 | Response to lipid | 194 | 980 | 0.007420702 |
| GO:0006656 | Phosphatidylcholine biosynthetic process | 14 | 36 | 0.011393706 |
| GO:0046474 | Glycerophospholipid biosynthetic process | 58 | 245 | 0.013038804 |
| GO:0044242 | Cellular lipid catabolic process | 57 | 241 | 0.013961285 |
| GO:0140353 | Lipid export from cell | 17 | 50 | 0.016831087 |
| GO:0071071 | Regulation of phospholipid biosynthetic process | 9 | 19 | 0.017155387 |
| GO:0009247 | Glycolipid biosynthetic process | 23 | 77 | 0.018930617 |
| GO:1903725 | Regulation of phospholipid metabolic process | 14 | 39 | 0.022394722 |
| GO:0006650 | Glycerophospholipid metabolic process | 76 | 353 | 0.031423921 |
| GO:0046890 | Regulation of lipid biosynthetic process | 43 | 179 | 0.031927887 |
| GO:0006688 | Glycosphingolipid biosynthetic process | 13 | 37 | 0.035422477 |
| GO:0046839 | Phospholipid dephosphorylation | 16 | 50 | 0.036744829 |
| GO:0046513 | Ceramide biosynthetic process | 21 | 73 | 0.039287038 |
| GO:0044283 | Small molecule biosynthetic process | 118 | 591 | 0.040600205 |
| GO:0051977 | Lysophospholipid transport | 3 | 3 | 0.040600205 |
| GO:0006636 | Unsaturated fatty acid biosynthetic process | 16 | 51 | 0.04256712 |
| **Epithelial-to-mesenchymal transition (EMT)** | | | | |
| GO:0007010 | Cytoskeleton organization | 342 | 1594 | 7.76E-08 |
| GO:0016477 | Cell migration | 337 | 1602 | 7.71E-07 |
| GO:0030334 | Regulation of cell migration | 221 | 977 | 9.62E-07 |
| GO:0030036 | Actin cytoskeleton organization | 182 | 769 | 7.02E-07 |
| GO:0001667 | Ameboidal-type cell migration | 114 | 446 | 5.48E-06 |
| GO:2000145 | Regulation of cell motility | 227 | 1038 | 7.82E-06 |
| GO:0048870 | Cell motility | 364 | 1806 | 1.47E-05 |
| GO:0048762 | Mesenchymal cell differentiation | 69 | 249 | 6.46E-05 |
| GO:0007015 | Actin filament organization | 116 | 481 | 6.69E-05 |
| GO:0030198 | Extracellular matrix organization | 90 | 355 | 0.00010718 |
| GO:0043062 | Extracellular structure organization | 90 | 356 | 0.000116952 |
| GO:0014031 | Mesenchymal cell development | 32 | 89 | 0.000126434 |
| GO:0060485 | Mesenchyme development | 79 | 313 | 0.0003894 |
| GO:0030335 | Positive regulation of cell migration | 127 | 563 | 0.000503307 |
| GO:0032970 | Regulation of actin filament-based process | 99 | 418 | 0.000547776 |
| GO:0030048 | Actin filament-based movement | 40 | 131 | 0.000587931 |
| GO:0032956 | Regulation of actin cytoskeleton organization | 89 | 376 | 0.001224505 |
| GO:0110053 | Regulation of actin filament organization | 69 | 288 | 0.004310403 |
| GO:0022617 | Extracellular matrix disassembly | 24 | 73 | 0.004771036 |
| GO:2000146 | Negative regulation of cell motility | 75 | 331 | 0.010790481 |
| GO:0030832 | Regulation of actin filament length | 43 | 169 | 0.012813033 |
| GO:0010631 | Epithelial cell migration | 72 | 318 | 0.013044323 |
| GO:0031532 | Actin cytoskeleton reorganization | 32 | 117 | 0.014639367 |
| GO:0090132 | Epithelium migration | 72 | 321 | 0.015890953 |
| GO:0010464 | Regulation of mesenchymal cell proliferation | 12 | 31 | 0.022389987 |
| GO:0010463 | Mesenchymal cell proliferation | 15 | 44 | 0.026975991 |
| GO:0008154 | Actin polymerization or depolymerization | 50 | 215 | 0.031720098 |
| GO:0010717 | Regulation of epithelial to mesenchymal transition | 27 | 100 | 0.033477921 |
| GO:0051495 | Positive regulation of cytoskeleton organization | 49 | 214 | 0.042533686 |
| GO:0030154 | Cell differentiation | 894 | 4552 | 3.57E-12 |
| GO:0034330 | Cell junction organization | 196 | 739 | 1.13E-11 |
| GO:0007155 | Cell adhesion | 360 | 1729 | 7.62E-07 |
| GO:0034329 | Cell junction assembly | 118 | 457 | 2.05E-06 |
| GO:0002009 | Morphogenesis of an epithelium | 131 | 550 | 3.39E-05 |
| GO:0045597 | Positive regulation of cell differentiation | 201 | 933 | 8.51E-05 |
| GO:0045595 | Regulation of cell differentiation | 332 | 1700 | 0.000465803 |
| GO:0098609 | Cell-cell adhesion | 225 | 1104 | 0.000724992 |
| GO:0098742 | Cell-cell adhesion via plasma-membrane adhesion molecules | 73 | 293 | 0.001036639 |
| GO:0060563 | Neuroepithelial cell differentiation | 15 | 41 | 0.014157117 |
| GO:0045216 | Cell-cell junction organization | 52 | 219 | 0.01912892 |
| GO:2000049 | Positive regulation of cell-cell adhesion mediated by cadherin | 8 | 17 | 0.028728563 |
| GO:0045785 | Positive regulation of cell adhesion | 120 | 600 | 0.03717823 |
| GO:0030155 | Regulation of cell adhesion | 177 | 927 | 0.039613277 |
| GO:0030855 | Epithelial cell differentiation | 147 | 756 | 0.040766976 |
| GO:2000047 | Regulation of cell-cell adhesion mediated by cadherin | 11 | 30 | 0.044580056 |
| **Calcium deposition** | | | | |
| GO:0032475 | Otolith formation | 7 | 7 | 0.000116302 |
| GO:0051592 | Response to calcium ion | 46 | 152 | 0.000225989 |
| GO:0032474 | Otolith morphogenesis | 8 | 11 | 0.001143837 |
| GO:0071895 | Odontoblast differentiation | 9 | 14 | 0.001517143 |
| GO:0046849 | Bone remodeling | 30 | 94 | 0.001939859 |
| GO:0002062 | Chondrocyte differentiation | 36 | 121 | 0.002058211 |
| GO:0071277 | Cellular response to calcium ion | 29 | 92 | 0.002950978 |
| GO:0001503 | Ossification | 97 | 435 | 0.004573393 |
| GO:0048709 | Oligodendrocyte differentiation | 31 | 104 | 0.004886633 |
| GO:0045453 | Bone resorption | 21 | 68 | 0.018898755 |
| GO:0048840 | Otolith development | 8 | 16 | 0.019926477 |
| GO:0032330 | Regulation of chondrocyte differentiation | 18 | 57 | 0.027409564 |
| GO:0045780 | Positive regulation of bone resorption | 9 | 21 | 0.033219926 |
| GO:0055074 | Calcium ion homeostasis | 104 | 508 | 0.033309662 |
| GO:0006816 | Calcium ion transport | 96 | 470 | 0.044500943 |
| GO:0060402 | Calcium ion transport into cytosol | 44 | 189 | 0.046075264 |

**Biological processes altered with time**

Figure S1 shows the comparative analysis of enriched pathways according to gene expression using GO database, at 4, 12, 17 and 25 weeks in culture.

**Figure S1.** **Enrichment of biological processes according to GO database between 4, 12, 17 and 25 weeks’ comparisons**. Bubble chart of the 20 most significantly enriched biological processes when comparing (A) 4 weeks (n=3) vs 12 weeks (n=3), (B) 4 weeks (n=3) vs 17 weeks (n=3), (C) 12 weeks(n=3) vs 17 weeks(n=3), (D) 12 weeks(n=3) vs 25 weeks (n=3), (E) 17 weeks(n=3) vs 25 weeks(n=3). Each process is represented by a bubble and indicated in the Y-axis. The size of the bubble shows the number of DEGs annotated to that GO term and its color, the significance of its enrichment (q-value). X-axis represents the enrichment ratio of each process (ratio between the number of DEGs annotated to the GO term and the total number of genes annotated that GO term).

**Gene expression of RPE markers**

Expression of genes associated with RPE identity was studied at 4, 12, 17 and 25 weeks in culture to evaluate the differentiation state of cells, showing increased expression of genes associated with RPE until 17 weeks in culture (Figure S2).

**Figure S2. Expression of RPE-specific genes over time. (A)** DEGs identified between 4 (n=3), 12 (n=3), 17 (n=3) and 25 (n=3) time points. Fold-changes and statistical significance were obtained by DESeq2 method. ns: q-value > 0.05; *: q-value < 0.05; **: q-value < 0.01; ***: q-value<0,001. **(B)** SCGs identified through linear and quadratic regression models. Equation of the regression curve is shown together with the associated coefficient of determination (R^2^) and p-value adjusted by Benjamini-Hochberg method.

**(A)**

|  | **Average Read Count** | | | | **Fold-Change (Significance)** | | |
| --- | --- | --- | --- | --- | --- | --- | --- |
| **Gene** | **4 weeks** | **12 weeks** | **17 weeks** | **25 weeks** | **12 vs 4 weeks** | **17 vs 4 weeks** | **25 vs 4 weeks** |
| *DCT* | 5710 | 1632 | 1856 | 16 | 0.32 (***) | 0.36 (***) | 0.003 (***) |
| *TYRP1* | 32714 | 14126 | 22838 | 176 | 0.48 (***) | 0.70 (*) | 0.01 (***) |
| *MITF* | 3727 | 1663 | 2264 | 452 | 0.49 (***) | 0.61 (***) | 0.15 (***) |
| *BEST1* | 17321 | 9344 | 17869 | 25 | 0.60 (ns) | 1.04 (ns) | 0.002 (***) |
| *RDH5* | 3108 | 1235 | 2185 | 146 | 0.44 (ns) | 0.71 (ns) | 0.057 (**) |
| *LRAT* | 683 | 353 | 1000 | 6 | 0.57 (ns) | 1.47 (ns) | 0.01 (**) |
| *RGR* | 923 | 1794 | 3676 | 5 | 2.14 (*) | 4.00 (***) | 0.01 (***) |
| *RPE65* | 133 | 114 | 390 | 1 | 0.94 (ns) | 2.94 (*) | 0.005 (***) |

**(B)**


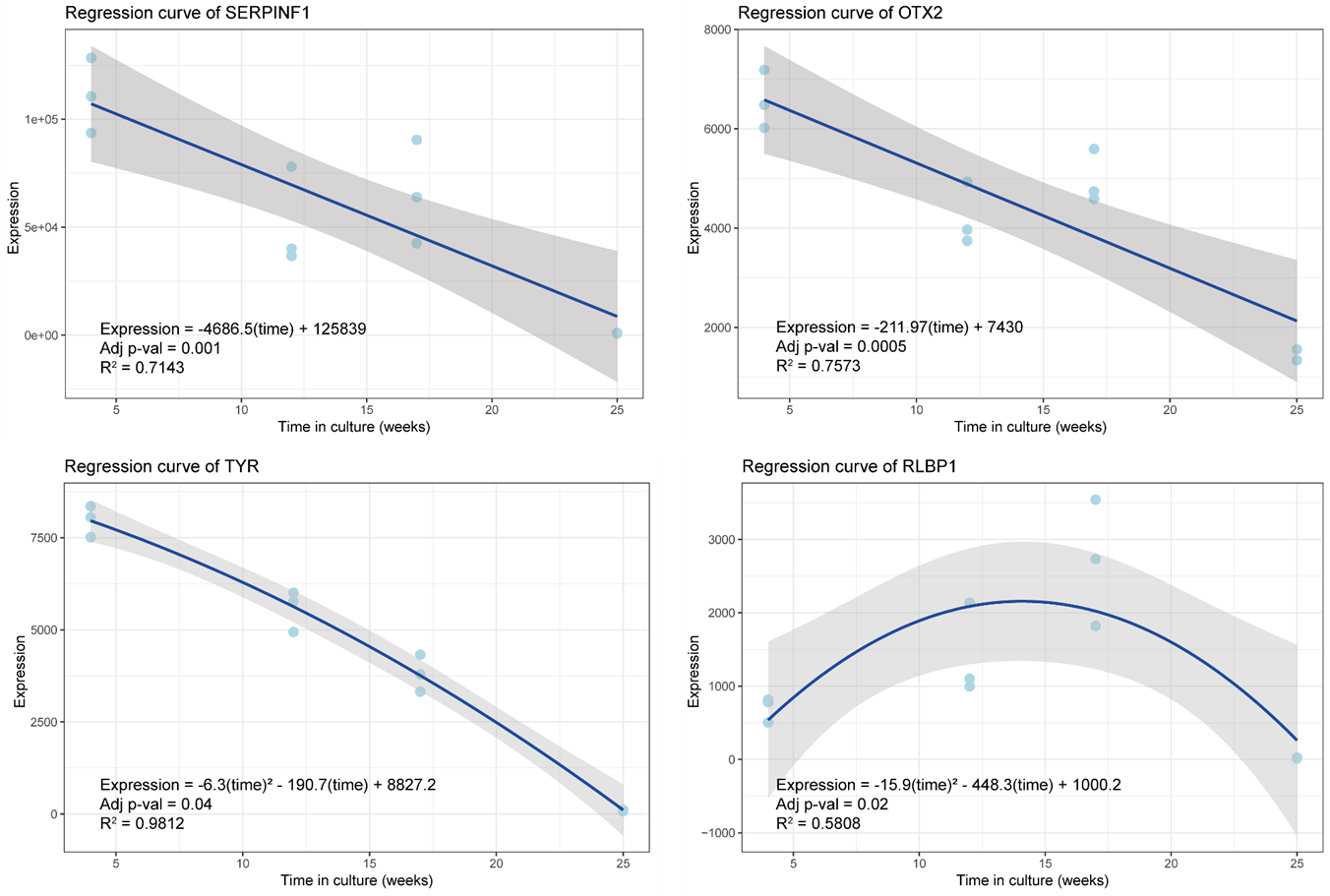


**Untargeted metabolomics analysis**

The Kruskal-Wallis mean comparison test identified 498 metabolites with significant differences among time points (p-value < 0.05) in positive ionization mode and 222 in negative ionization mode. Applying an additional for metabolites with a fold-change > 2, reduced the list to 283 features in positive ionization mode and 100 features in negative ionization mode. A heat map of hierarchical clustering analysis for these differential metabolites is presented in Figure S3, showing comparisons for 25-week-old cells against 4-, 12-, and 17-week-old cells. Tentative identification of these compounds using ID browser resulted in 185 distinct molecules in positive ionization mode and 68 in negative ionization mode. Normalized averaged data for each tentatively identified compounds are provided in Table S2 and S3 of Additional File 2.

**Figure S3**. Heatmap of hierarchical clustering analysis of differential molecular features in 25-week-old (n=3 biological replicates, each measured 3 times) RPE cells versus 4- (n=3 biological replicates, each measured 3 times), 12- (n=3 biological replicates, each measured 3 times) and 17-week-old (n=3 biological replicates, each measured 3 times) cells. (A) in positive ionization mode, (B) in negative ionization mode. Significantly different compounds were identified using the Kruskal-Wallis test with Benjamini-Hochberg correction (adjusted p-value < 0.05 and fold-change > 2).
